# *HTLV-1 bZIP Factor* Induces T-Cell Lymphoma and Systemic Inflammation *In Vivo*

**DOI:** 10.1371/journal.ppat.1001274

**Published:** 2011-02-10

**Authors:** Yorifumi Satou, Jun-ichirou Yasunaga, Tiejun Zhao, Mika Yoshida, Paola Miyazato, Ken Takai, Kei Shimizu, Koichi Ohshima, Patrick L. Green, Naganari Ohkura, Tomoyuki Yamaguchi, Masahiro Ono, Shimon Sakaguchi, Masao Matsuoka

**Affiliations:** 1 Laboratory of Virus Control, Institute for Virus Research, Kyoto University, Kyoto, Japan; 2 Department of Pathology, Kurume University School of Medicine, Kurume, Japan; 3 Center for Retrovirus Research and Departments of Veterinary Biosciences and Molecular Virology, Immunology and Medical Genetics, The Ohio State University, Columbus, Ohio, United States of America; 4 Department of Experimental Pathology, Institute for Frontier Medical Sciences, Kyoto University, Kyoto, Japan; University of Geneva, Switzerland

## Abstract

Human T-cell leukemia virus type 1 (HTLV-1) is the causal agent of a neoplastic disease of CD4^+^ T cells, adult T-cell leukemia (ATL), and inflammatory diseases including HTLV-1 associated myelopathy/tropical spastic paraparesis, dermatitis, and inflammatory lung diseases. ATL cells, which constitutively express CD25, resemble CD25^+^CD4^+^ regulatory T cells (T_reg_). Approximately 60% of ATL cases indeed harbor leukemic cells that express FoxP3, a key transcription factor for T_reg_ cells. HTLV-1 encodes an antisense transcript, *HTLV-1 bZIP factor* (*HBZ*), which is expressed in all ATL cases. In this study, we show that transgenic expression of HBZ in CD4^+^ T cells induced T-cell lymphomas and systemic inflammation in mice, resembling diseases observed in HTLV-1 infected individuals. In *HBZ*-transgenic mice, CD4^+^Foxp3^+^ T_reg_ cells and effector/memory CD4^+^ T cells increased *in vivo*. As a mechanism of increased T_reg_ cells, HBZ expression directly induced *Foxp3* gene transcription in T cells. The increased CD4^+^Foxp3^+^ T_reg_ cells in HBZ transgenic mice were functionally impaired while their proliferation was enhanced. HBZ could physically interact with Foxp3 and NFAT, thereby impairing the suppressive function of T_reg_ cells. Thus, the expression of HBZ in CD4^+^ T cells is a key mechanism of HTLV-1-induced neoplastic and inflammatory diseases.

## Author Summary

Human T-cell leukemia virus type 1 (HTLV-1) is the first retrovirus that is associated with human diseases including an aggressive leukemia derived from CD4^+^ T cells, adult T-cell leukemia (ATL), and chronic inflammatory diseases of the central nervous system, lung, or skin. However, it remains to be elucidated how HTLV-1 induces these diseases. A viral gene, *tax*, has been considered as a critical player in HTLV-1 pathogenesis, yet Tax expression is frequently lost in ATL cells. Another viral gene, *HBZ*, is constitutively expressed in both HTLV-1 infected cells and ATL cells. However, it remains unknown how HBZ functions in the HTLV-1-related diseases. We show here that the *HBZ* induced T-cell lymphoma and chronic inflammation *in vivo* similar to those in HTLV-1 infected individuals, indicating an important role of *HBZ* in HTLV-1 associated human diseases. As observed in HTLV-1 infected individuals, effector/memory and regulatory CD4^+^ T cells were increased in the *HBZ*-transgenic mice. Further, HBZ could interact with host transcription factors, Foxp3 and NFAT, leading to dysregulation of T_reg_ function. The T_reg_ dysregulation induced by HBZ is thought to be a critical mechanism of the HTLV-1 pathogenesis. This study sheds light on the HTLV-1 associated pathogenesis and provides an important clue to prevent or treat the human diseases.

## Introduction

Human T-cell leukemia virus type 1 (HTLV-1) was the first human retrovirus associated with human diseases including adult T-cell leukemia (ATL) [1], [2] and HTLV-1 associated myelopathy/tropical spastic paraparesis (HAM/TSP)[3], [4]. One of the virological attributes of HTLV-1 is that it transmits mainly by cell-to-cell contact [5], [6]. Therefore, HTLV-1 induces the proliferation of infected CD4^+^ T cells to increase further transmission [7]. HTLV-1 encodes several regulatory and accessory genes in the pX region located between the *env* gene and the 3′ LTR [7], [8]. Among the viral genes, *tax* possesses *in vitro* transforming activity and can induce cancers in transgenic (Tg) animals via its pleiotropic actions [9], [10]. Yet the expression of Tax is frequently disrupted in ATL [7]. In contrast, the *HTLV-1 bZIP factor* (*HBZ*) gene, which is encoded in the minus strand of the HTLV-1 genome [11], [12], is transcribed in all ATL cases [13]. Recently, it has been reported that APOBEC3G generates nonsense mutations in all HTLV-1 genes except *HBZ* [14], suggesting that the *HBZ* gene is indispensable for the growth and/or survival of ATL cells and HTLV-1 infected cells. The *HBZ* gene product promotes the proliferation of ATL cells [13], [15]. Further, *HBZ* mRNA expression in HAM/TSP patients was well correlated with disease severity [16]. These findings suggest that *HBZ* has a critical role in the development of ATL and HAM/TSP.

It has been shown that ATL cells functionally and phenotypically resemble Foxp3^+^ CD25^+^CD4^+^ regulatory T (T_reg_) cells, which control immune responses against self- and non-self-antigen [17]. ATL cells constitutively express CD25 and scarcely produce interleukin-2 (IL-2)[18], [19]. Furthermore, two thirds of ATL cases harbor leukemic cells expressing FoxP3 [20], [21], a key transcription factor for the generation and function of T_reg_ cells [22], [23], [24]. In HTLV-1 carriers, HTLV-1 provirus is detected mainly in CD4^+^ effector/memory T cells and T_reg_ cells [25], [26], [27]. Thus, HTLV-1 favors T_reg_ cells and effector/memory T cells *in vivo*, and transforms them. However, how HTLV-1 targets these T cell subpopulations remains to be elucidated.

In this study, we show that transgenic expression of HBZ increases Foxp3^+^ T_reg_ cells and effector/memory T cells, leading to development of T-cell lymphomas and systemic inflammatory diseases. In addition, the suppressive function of T_reg_ cells is severely impaired in HBZ transgenic mice. At the molecular level, we show that HBZ interacts with Foxp3 and NFAT, interrupting the function of each molecule, and leading to the deregulation of Foxp3-mediated transcriptional control of the genes associated with T_reg_ functions. These results indicate that HBZ plays a critical role in neoplastic and inflammatory diseases arising from HTLV-1 infection.

## Results

### HBZ transgenic mice spontaneously develop inflammatory lesions in the skin and lung

**Figure 1:**
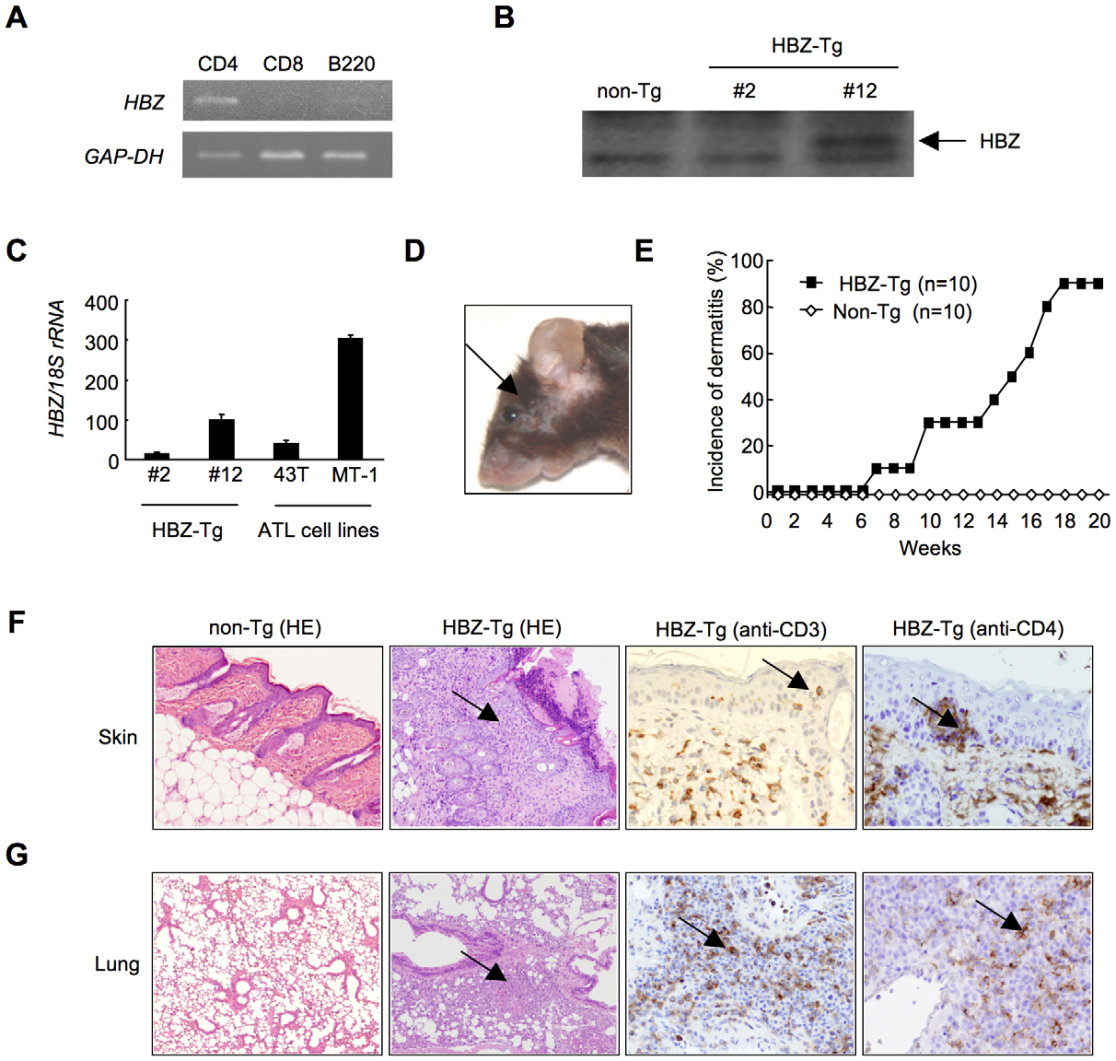
*HBZ*-Tg mice spontaneously develop inflammatory diseases in skin and lung. (A) Cell-type specific transcription of the transgene in line 12 was confirmed by RT-PCR in each sorted cell population. (B) The expression of HBZ protein in CD4^+^ splenocytes was confirmed by Western blotting. (C) Transcripts of the *HBZ* gene in CD4^+^ splenocyte of *HBZ*-Tg mice or ATL cell lines were quantified by real time PCR. ATL-43T and MT-1 are derived from ATL cells. (D) An *HBZ*-Tg mouse with typical skin symptom (Arrow indicates skin lesion). (E) The incidence of dermatitis in *HBZ*-Tg (line 12) and non-Tg mice. (F and G) Histological findings of the skin and the lung in *HBZ*-Tg mice. Lymphocytes massively infiltrated the dermis and epidermis (F) and the alveolar septum (G) (Arrows present infiltration of lymphocytes). Infiltration of CD3^+^, CD4^+^ T cells into these tissues was shown by immunohistochemistry compared with non-Tg mice as control.

Since HTLV-1 mainly infects CD4^+^ T cells *in vivo*, we generated Tg mice expressing the *HBZ* gene under the control of the murine *CD4*-specific promoter/enhancer/silencer (Figure S1) [13]. We analyzed the *HBZ* transgenes (Figure S1) and their expression in the three lines generated. *HBZ* gene expression was specifically detected in CD4^+^ T cells (Figure 1A). HBZ protein was also detected in these transgenic mice (Figure 1B). The level of *HBZ* gene transcripts in line 12 was the most abundant but similar to that of endogenous expression of the *HBZ* gene in ATL cell lines (Figure 1C). Therefore, unless specifically described, we used line 12 in this study. Notably, the majority of *HBZ*-Tg mice developed skin lesions by 18 weeks of age, in contrast with no disease in non-transgenic littermates (non-Tg mice) (Figure 1, D and E). Histological analyses revealed infiltration of CD3^+^CD4^+^ T cells into the dermis and epidermis, and also the alveolar septa of the lung (Figure 1, F, G and S2), whereas no obvious evidence of inflammation in other tissues, including liver, kidney, muscle, heart, stomach, spinal cord, intestines and brain. Since massive infiltration of lymphocytes in the skin and lung was observed in line 9 and 12, but not in line 2, level of HBZ expression is likely associated with these phenotypes. Thus, *HBZ*-Tg mice spontaneously developed dermatitis and alveolitis. Similar lesions have been observed in HTLV-1 carriers, especially in those harboring large numbers of infected cells [28], [29].

### *HBZ*-Tg mice develop T-cell lymphoma after a long latent period

**Figure 2:**
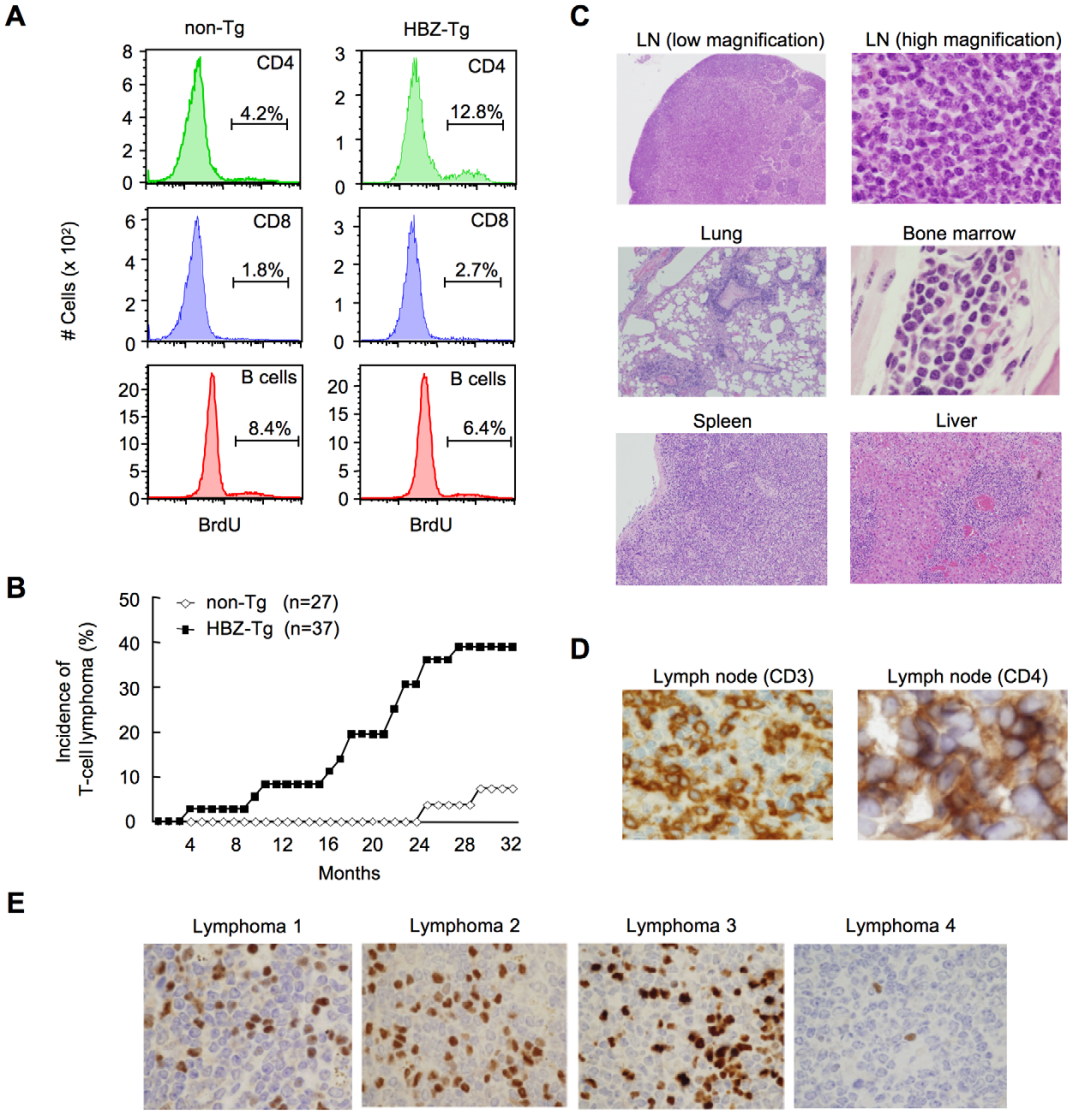
*HBZ-*Tg mice develop T-cell lymphoma after a long latent period. (A) BrdU was injected into mice twice a day for three days, and splenocytes were stained with antibodies to BrdU, CD4, CD8, and B220. (B) Incidence of T-cell lymphoma in *HBZ*-Tg mice was statistically significant compared with that in non-Tg mice (*P*<0.001 by the logrank test). (C) Pleomorphic lymphoma in the cervical lymph node in a representative *HBZ*-Tg mouse. Infiltrations of lymphoma cells into lung, bone marrow, spleen and liver are also shown. (D) Expression of CD3 and CD4 in lymphoma cells was shown by immunohistochemical staining. (E) Immunohistochemical staining for Foxp3 in primary lymphomas of *HBZ-*Tg mice.

To study the growth-promoting activity of the *HBZ* gene, we assessed the proliferation of CD4^+^ T cells in *HBZ*-Tg mice by incorporation of bromodeoxyuridine (BrdU), and found that the proliferation was three fold-higher than in non-Tg mice, whereas the proliferation of CD8^+^ T cells or B cells was not altered (Figure 2A, Table S1A). Transgenic expression of *HBZ* enhances the *in vivo* proliferation of mouse T cells, as ectopic expression of *HBZ* enhances the proliferation of human T cells [13], [15]. It is known that HTLV-1 transforms CD4^+^ T cells after a long latent period in a fraction of asymptomatic carriers [7]. Analogous to the development of ATL in humans, 14 of 37 (37.8%) *HBZ*-Tg mice of all three-founder lines developed T-cell lymphomas after 16 months, in contrast with 2 of 27 non-Tg mice (7.4%) (*P*<0.001 by the logrank test) (Figure 2B). In some transgenic mice, lymphoma cells infiltrated various organs including the lung, bone marrow, spleen and liver (Figure 2C). All of the lymphomas in *HBZ*-Tg mice were CD3^+^ and CD4^+^ by immunohistochemical analyses when examined before the mice became moribund (Figure 2D). Lymphoma cells also expressed αβT cell receptors on their surfaces (Figure S3). Monoclonal proliferation of these lymphoma cells was shown by single strand conformation polymorphism in Vγ2-Jγ1 junction region of T cell receptor γchain gene (Figure S4). Notably, the primary lymphoma cells expressed Foxp3 at various intensities in the majority of cases (Figure 2E, Table 1), exhibiting a similar FoxP3 staining pattern to that in lymph nodes in human ATL cases (Figure S5). Thus, the T-cell lymphomas in *HBZ*-Tg mice phenotypically resemble ATL, suggesting that HBZ promotes proliferation of CD4^+^ T cells and predisposes expressing cells to transform in due course.

**Table 1:** Characteristics of lymphomas in *HBZ*-Tg and non-Tg littermates.

			Latency		IHC		
Genotype	Strain	ID	(Months)	B220	CD3	Foxp3	Phenotype
non-Tg			24	-	-	-	non-T, non-B
(2/27)			24	+	-	-	B
			26	+	-	-	B
			13	-	-	-	non-T, non-B
			27	-	+	+	T
			32	-	+	+	T
HBZ-Tg	#2	1	11	-	+	++	T
(14/37)	(3/14)	2	19	-	+	-	T
		3	24	-	+	+	T
	#9	1	23	+	+	+	T
	(4/5)	2	18	+	+	++	T
		3	26	+	+	+	T
		4	29	-	+	+	T
	#12	1	23	-	±	++	T
	(7/18)	2	19	-	+	+	T
		3	5	-	+	+++	T
		4	10	-	+	++	T
		5	27	+	+	-	T
		6	24	-	±	++	T
		7	18	-	+	+	T

Mice that died or became immobilized were subjected to autopsy. Tissue samples were surgically removed, fixed in 10% formalin in phosphate buffer, embedded in paraffin and stained with hematoxylin and eosin for histopathological examination. Tissue samples with lymphoma were subjected to immunohistochemical analysis (IHC) using monoclonal antibodies for CD3 (500A2), B220 (RA3-6B2), and Foxp3 (FJK-16s). The phenotype of lymphomas was determined based on CD3 and B220 expression. The degree of Foxp3 expression in lymphomas was evaluated by immunohistochemistry. (+, 1–9%; ++, 10–20%; +++, more than 20%) Frequency of T-cell lymphoma of each line is shown as follows; (number of T-cell lymphoma/number of total observed mice).

### Increased effector/memory and regulatory CD4^+^ T cells in *HBZ*-Tg mice

**Figure 3:**
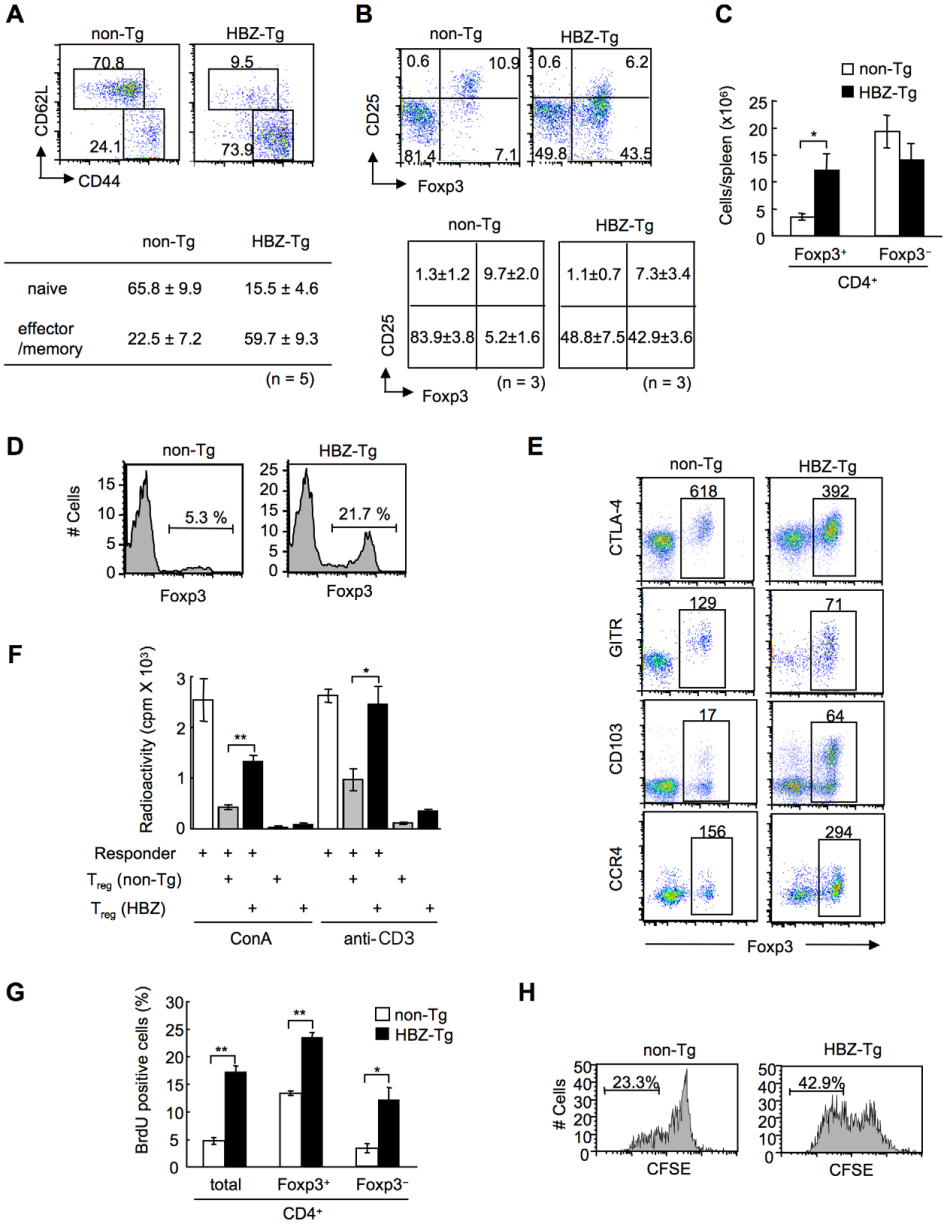
Transgenic expression of HBZ in CD4^+^ T cells increases Foxp3^+^ T_reg_ cells with impaired suppressive function. (A and B) Mouse splenocytes were stained with the indicated antibodies, and analyzed by flow cytometry. Representative dot plots gated on the CD4^+^ population are shown. For these experiments, *HBZ*-Tg mice without any symptoms were used. Tables show the mean ± SD (n = 5 for A, n = 3 for B). (C) The absolute number of Foxp3^+^ or Foxp3^−^ CD4^+^ T cells in *HBZ*-Tg and non-Tg mice. The results shown are the mean ± SD (n  =  3). (D) Flow cytometric analysis for the Foxp3 expression in CD4 single positive thymocytes. Representative dot plots gated on the CD4 single positive population are shown from three independent analyses. (E) Flow cytometric analyses of CD4^+^ T cells for T_reg_ related molecules. Numbers in dot plots indicate mean fluorescence intensity (MFI) of each molecule in the rectangular gates. (F) Suppressive activity of T_reg_ cells from *HBZ-* or non-Tg mice on T-cell proliferation. Sorted Foxp3^+^ T cells were cultured with CD4^+^CD25^−^ cells of non-Tg mice as responder cells for 72 h with ConA or soluble anti-CD3 antibody and x-irradiated antigen presenting cells (APCs), and [^3^H] thymidine incorporation during the last 6 hours was measured. Results are means ± SD for triplicate cultures. (G) *In vivo* BrdU incorporation in total CD4^+^, Foxp3^+^CD4^+^, or Foxp3^−^CD4^+^ T cells. The results shown are the mean ± SD (n = 3). (H) Sorted Foxp3^+^ cells were labeled with CFSE and cultured with anti-CD3 antibody and x-irradiated APCs. After 96 hours, the cells were stained with anti-Foxp3, and CFSE dilution was analyzed for Foxp3^+^ cells. *, *P*<0.01; **, *P*<0.001 by two-tailed Student *t*-test.

To study the cellular basis of the lymphomagenesis and inflammation in *HBZ*-Tg mice, we analyzed the phenotype and function of T cells, especially T_reg_ cells, in 3-month-old *HBZ-*Tg line 12 mice before their pathological manifestations. CD44^high^ CD62L^low^ effector/memory CD4**^+^** T cells increased in *HBZ*-Tg mice (Figure 3A). CD4 single positive T cells also increased in the thymus (Figure S6). Further, not only the ratio but also the absolute number of Foxp3**^+^** T cells was markedly increased in *HBZ-*Tg mice compared with non-Tg mice, while the numbers of Foxp3**^−^** T cells were equivalent (Figure 3, B and C). Increased T_reg_ cells were also observed in thymus, lymph node and peripheral blood mononuclear cells (Figure 3D and Figure S7). We also observed the increased T_reg_ cells and effector/memory T cells in the *HBZ*-Tg line 2 (Figure S8), which showed quite lower expression of HBZ than line 12 (Figure 1C). The proportion of T_reg_ cells in skin and lung was rather low compared with that in spleen (Figure 3B and S2), indicating that Foxp3^−^ T cells are predominant in the infiltrating T cells.

This result indicates that transgenic expression of HBZ induces systemic inflammation despite an increase in Foxp3^+^ T_reg_ cells. It has been reported that IL-2 is critical in the homeostasis of T_reg_ cells [30]. To study mechanisms by which HBZ expression increases T_reg_ cells, we analyzed IL-2 production in the CD4^+^ T cells of *HBZ*-Tg mice after stimulation by PMA and ionomycin. IL-2 production was not augmented in either the Foxp3^+^ or Foxp3^−^ populations from *HBZ*-Tg mice (Figure S9), indicating that the increase in the number of T_reg_ cells was not due to enhanced IL-2 production.

Previous studies showed that Tax is a critical viral protein for the pathogenesis of HTLV-1. Therefore, we generated Tax transgenic (*tax*-Tg) mice using the same promoter/enhancer/silencer. In the *tax*-Tg mice, we did not observe increased effector/memory T cells or T_reg_ cells (Figure S10). Thus, this increase in effector/memory T cells and T_reg_ cells was specific to HBZ and not associated with similar transgenic expression of *tax* in this transgenic model system.

We next analyzed the phenotype and function of the increased Foxp3^+^ T_reg_ cells in *HBZ*-Tg mice. CD4^+^Foxp3^+^ T cells of *HBZ*-Tg mice expressed T_reg_-associated molecules, such as cytotoxic T-lymphocyte associated antigen-4 (CTLA-4), glucocorticoid-induced TNF receptor family-related-protein (GITR), CD103, and CD25 [31]; yet the expression levels of CTLA-4, GITR and CD25 were lower than those of Foxp3^+^ T cells in non-Tg mice (Figure 3, B and E, Table S1B). In contrast, both Foxp3^+^ and Foxp3^−^ CD4^+^ T cells of *HBZ*-Tg mice expressed CCR4 and CD103 at higher levels than those in non-Tg mice, suggesting that this might contribute to the migration and infiltration of *HBZ*-Tg CD4^+^ T cells into the skin (Figure 1F) [32], [33]. Further, it is of note that the *in vitro* suppressive function of *HBZ*-Tg T_reg_ cells was severely impaired. When CD4^+^GITR^high^ T cells, which were >90% Foxp3^+^ [23], from *HBZ*-Tg or non-Tg mice were co-cultured with CD4^+^CD25^−^ T cells from wild-type mice and stimulated with Con A or anti-CD3 antibody, *HBZ*-Tg T_reg_ cells were much less suppressive (Figure 3F). These results indicate that HBZ expression increases functionally impaired T_reg_ cells.

Next, we assessed the proliferation of CD4^+^ T cells in *HBZ*-Tg mice. BrdU incorporation of Foxp3^+^ as well as Foxp3^−^CD4^+^ T cells from *HBZ*-Tg mice was also significantly higher than those in non-Tg mice (Figure 3G). In general, proliferation of T_reg_ cells in response to mitogenic stimulation is suppressed *in vitro*. However, Foxp3^+^ T cells from *HBZ*-Tg mice proliferated more vigorously *in vitro* in response to anti-CD3 antibody than did non-Tg Foxp3^+^ T cells (Figure 3H). Thus, transgenic expression of HBZ in CD4^+^ T cells induces the expansion of Foxp3^+^ T_reg_ cells, yet impairs their suppressive function.

### HBZ directly induces Foxp3 expression in a CD4^+^ T-cell intrinsic manner

**Figure 4:**
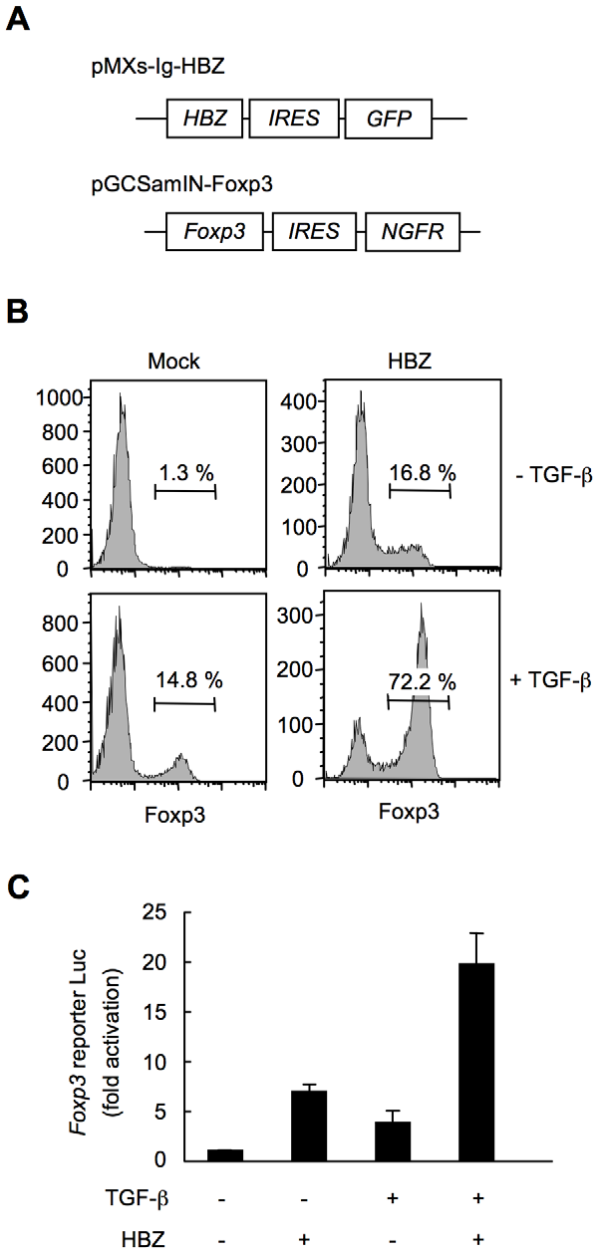
HBZ directly induces Foxp3 expression in CD4^+^ T cells. (A) Schematic diagrams of retrovirus vectors used in this study. (B) Mouse CD4^+^CD25^−^ T cells transduced with retrovirus vector encoding HBZ or empty vector with or without TGF-β were stained with anti-Foxp3 antibody and analyzed by flow cytometry. (C) To study the effect of HBZ on promoter activity of the *Foxp3* gene, EL4 cells were transfected with Foxp3 reporter plasmid and/or HBZ expressing plasmid. Representative data shown are firefly luciferase activities normalized to those of renilla luciferase (mean ± SD).

To study whether HBZ increases Foxp3^+^ T_reg_ cells in a cell intrinsic manner, we expressed HBZ in naive CD4^+^ T cells *in vitro* using a retrovirus vector (Figure 4A). Interestingly, HBZ induced Foxp3 expression in 16.8% of HBZ expressing T cells, which is a similar enhancement to that due to TGF-β treatment (14.8%). The expression was markedly augmented in HBZ expressing T cells treated with TGF-β (72.2%) (Figure 4B). A reporter assay using the enhancer and promoter of the *Foxp3* gene [34] demonstrated that HBZ induced transcription of the *Foxp3* gene (Figure 4C), which was enhanced in the presence of TGF-β. Thus, HBZ-induced Foxp3 expression could be a mechanism for the increase of Foxp3^+^ T cells in *HBZ*-Tg mice.

### HBZ physically interacts with Foxp3

**Figure 5:**
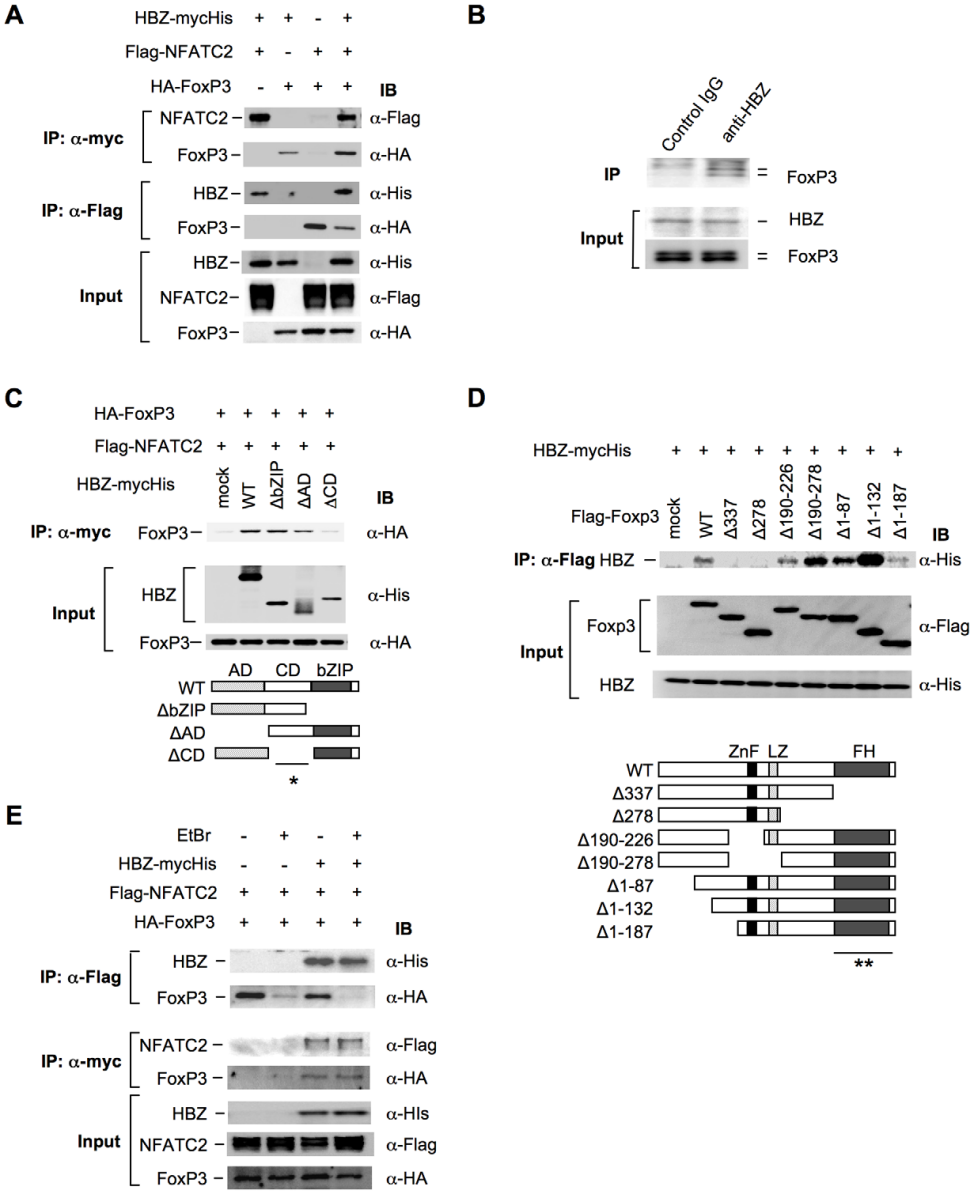
HBZ physically interacts with Foxp3 and NFAT. (A) The expression vectors of the indicated proteins were co-transfected into 293FT cells, and their interactions were analyzed by immunoprecipitation (IP). (B) Nuclear extract of ATL-43T cells was subjected to IP with anti-HBZ antibody or control IgG, and detected by anti-FoxP3 antibody. (C and D) The interactions of HBZ and Foxp3 were analyzed by IP using HBZ mutants (C) or Foxp3 mutants (D). A schematic diagram of Foxp3 mutants is shown. ZnF, zinc finger; LZ, leucine zipper; FH, forkhead domain. Asterisks (* or **) show responsible region for each molecular interaction. (E) The interactions among HBZ, Foxp3 and NFATc2 were analyzed with or without EtBr.

Previous studies have shown that Foxp3 controls T_reg_ function by cooperating with transcription factors including NFAT [35] and AML-1/Runx1[36]. Impaired interactions of Foxp3 with these factors not only alter the suppressive function of T_reg_ cells but also suppress the expression of T_reg_ associated molecules, such as CD25, CTLA-4, and GITR [23], [35], [36], [37], which is similar to the phenotype observed in *HBZ*-Tg mice (Figure 3, B and E). These findings prompted us to assess the possibility that HBZ might be involved in Foxp3-dependent transcriptional regulation. To address this, we first examined direct interaction among HBZ, NFAT and Foxp3. Immunoprecipitation experiments showed that HBZ physically interacted with both NFAT and Foxp3 (Figure 5A). Moreover, to study the interaction of endogenous HBZ and Foxp3, we performed immunoprecipitation using ATL-43T, a Foxp3-expressing ATL cell line. An anti-HBZ antibody co-precipitated endogenous Foxp3 in the ATL-43T cells, demonstrating that the interaction occurs not only in an enforced over-expressed state but also under physiological conditions (Figure 5B). It has been previously reported that human FoxP3 protein migrates as a doublet, which coincides with this result [38]. Analyses using HBZ deletion mutants showed that the central domain of HBZ interacted with Foxp3 (Figure 5C). Experiments with Foxp3 deletion mutants revealed that HBZ interacted with the forkhead (FH) domain of Foxp3 (Figure 5D). It has been reported that the region between the forkhead domain and the leucine zipper domain of Foxp3 interacted with AML-1 [36]. HBZ did not inhibit the binding between Foxp3 and AML-1 nor the suppressive effect of Foxp3 on AML-1-mediated transcription from the IL-2 gene promoter (Figure S11), indicating that HBZ does not influence Foxp3/AML1 mediated gene regulation.

To study whether HBZ independently interacts with Foxp3 and NFAT or, alternatively, if these molecules form a ternary complex, we studied the effect of the DNA intercalator ethidium bromide (EtBr) on their interactions. As shown in Figure 5E, the interactions of HBZ with Foxp3 or NFAT were not affected by EtBr while the interaction between NFAT and Foxp3 was diminished by EtBr as reported previously [35]. These findings suggest that the interactions of HBZ with NFAT and Foxp3 are independent of DNA while the interaction between NFAT and Foxp3 requires the presence of DNA.

### HBZ inhibits Foxp3-mediated CTLA-4 and GITR expression in CD4^+^ T cells *in vitro*

**Figure 6:**
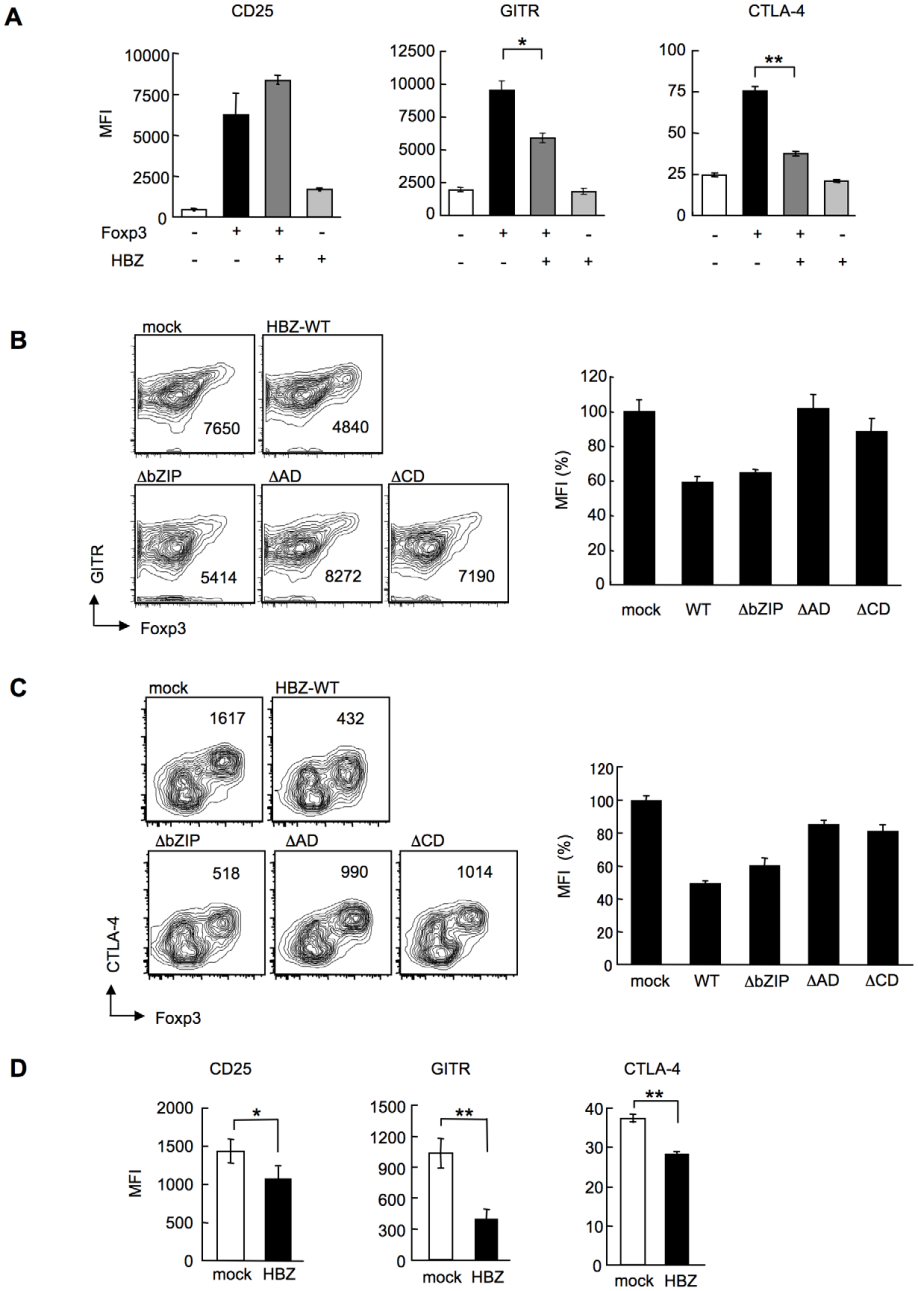
HBZ inhibites Foxp3-mediated CTLA-4 and GITR expression *in vitro*. (A) Mouse CD4^+^CD25^−^ T cells co-transduced with the retroviral vectors were stained with the indicated antibodies. Mean fluorescence intensity (MFI) of CD25, GITR, and CTLA-4 in GFP/NGFR double-positive cells are shown as mean ± SD. for triplicate culture. *, *P*<0.01; **, *P*<0.001 by two-tailed Student *t*-test. (B and C) CD4^+^CD25^−^ T cells transduced with the pMXs-Ig vector encoding wild-type or mutant HBZ, and pGCSamIN-Foxp3 vector were stained with anti-GITR (B) or anti-CTLA-4 (C) antibody in addition to anti-NGFR antibody, and then analyzed by flow cytometry. *Left*, numbers in density plots indicate MFI of GITR (B) or CTLA-4 (C) in GFP/NGFR double-positive cells. Representative data from three independent experiments are shown. *Right*, relative MFI of wild type or mutated HBZ compared to mock transduced cells was shown as mean ± SD (n = 3). (D) HBZ transduction in Foxp3^+^ T_reg_ cells inhibited the endogenous expression of T_reg_ associated molecules. Mean fluorescence intensity (MFI) of CD25, GITR, and CTLA-4 in CD4^+^Foxp3^+^NGFR^+^ cells are shown as mean ± SD. for triplicate culture. *, *P*<0.05; **, *P*<0.01 by two-tailed Student *t*-test.

In *HBZ*-Tg mice, the expression of T_reg_-associated molecules including CTLA-4, GITR and CD25 was suppressed when compared with their expression in T_reg_ cells from non-Tg mice (Figure 3B and E). This finding may account for the impaired function of T_reg_ cells since these molecules, in particular CTLA-4, play a critical role in T_reg_-mediated suppression [39]. To further study the effect of HBZ on the expression of T_reg_-associated molecules, we transduced HBZ along with Foxp3 into naive CD4^+^ T cells *in vitro* using retrovirus vectors (Figure 4A). HBZ expression suppressed Foxp3-induced GITR and CTLA-4 expression whereas it did not inhibit CD25 expression (Figure 6A). Expression of HBZ alone increased CD25 expression (Figure 6A), which might obscure the suppressive effect of HBZ under these conditions. Suppression of GITR and CTLA-4 expression required both the activation and the central domains of HBZ (Figure 6, B and C), which correspond to the binding sites of HBZ to Foxp3 (Figure 5C) and NFAT (Figure S12). Since both Foxp3 and NFAT are critical for T_reg_ function [35], it is likely that HBZ suppresses the expression of GITR and CTLA-4 by interacting with Foxp3 and NFAT and thereby interfering with their transcriptional regulation in T_reg_ cells. To examine suppressive effect of HBZ on expression of GITR, CTLA-4 and CD25, we isolated T_reg_ cells from wild type mice and expressed HBZ using retroviral vectors. As shown in Figure 6D, HBZ suppressed endogenous expression of CD25, GITR and CTLA-4 in T_reg_ cells, confirming that HBZ is responsible for suppressed expression of these molecules.

## Discussion

HTLV-1 targets CD4^+^ T cells; cell central to immune regulation. In contrast to human immunodeficiency virus, which destroys CD4^+^ T cells, HTLV-1 increases its copy number by inducing clonal proliferation of infected cells *in vivo* [40], [41]. Since HTLV-1 spreads mainly by cell-to-cell transmission [5], increased number of infected cells facilitates transmission of HTLV-1 to new cells. Recent studies showed that glucose transporter 1, heparan sulfate proteoglycans and neuropilin-1 are important for the entry of HTLV-1[42], [43], [44], consistent with the finding that this virus can infect a variety of cell types [45], [46]. However, HTLV-1 provirus is detected mainly in the regulatory and effector/memory CD4^+^ T cells of HTLV-1 carriers (Figure S13) [25], [26], [27], which indicates that HTLV-1 favors these specific subpopulations of CD4^+^ T cells. These findings suggest that HTLV-1 induces proliferation and/or facilitates survival of the regulatory and effector/memory CD4^+^ T cells. The mechanism(s) by which HTLV-1 targets T_reg_ cells, however, remained unclear until now. In this study, we showed that HBZ could enhance transcription of the *Foxp3* gene, and also promote proliferation of Foxp3^+^CD4^+^ T cells in transgenic mice, indicating that HBZ enhances both the generation and proliferation of Foxp3^+^ T cells. Impaired Foxp3 function is associated with proliferation of T_reg_ cells [37], so the HBZ-mediated T_reg_ dysfunction may also contribute to T_reg_ proliferation in addition to direct growth proliferation by the HBZ transcript [13]. Another possible explanation is that T_reg_ cells might be more susceptible to HTLV-1 infection, since T_reg_ cells proliferate vigorously *in vivo* presumably by recognizing self-antigen and commensal microbes [47]. With these strategies, HTLV-1 likely targets this specific T-cell population as its host, which might be beneficial for their survival.

As mechanisms of the HBZ-mediated effect on Foxp3 functions, we demonstrated that HBZ physically interacted with Foxp3 and impaired its function *in vitro*. HBZ lacking the Foxp3-binding region showed a slight inhibitory effect on Foxp3 function, indicating that direct interaction between HBZ and Foxp3 is, at least in part, responsible for suppression. The results of immunoprecipitation analyses using Foxp3 mutants showed that the forkhead domain of Foxp3 was responsible for the molecular interaction between HBZ and Foxp3. Since the forkhead domain is the DNA-binding domain of Foxp3 [17], HBZ might inhibit the transcriptional function of Foxp3 by interfering with the DNA binding activity. Foxp3 play a key role in the function and homeostasis of T_reg_ cells [22], [23], [24], indicating that HBZ-mediated dysfunction of Foxp3 contributes to impaired T_reg_ function in *HBZ*-Tg mice. This impaired T_reg_ function allows non-regulatory T cells to become hyper-reactive to commensal microbes and self-antigens, provoking enhanced proliferation of non-regulatory T cells and T cell-mediated autoimmune/inflammatory disease. These data collectively suggest that the viral protein HBZ hijacks the transcriptional machinery of host T_reg_ cells leading to inflammatory disorders in the host. Conversely, Tax, another HTLV-1 protein, has been reported to suppress FoxP3 expression in human T cells *in vitro* [48]. Therefore, it is likely that both viral proteins target Foxp3 albeit with apparently different effects. Considering that HBZ is consistently expressed while Tax expression is sporadic, Tax might control excess expression of Foxp3 in HTLV-1 infected cells.

In this study, we demonstrated that the characteristics of CD4^+^ T cells in *HBZ*-Tg mice resemble those of human ATL cells or HTLV-1 infected cells in carriers. First, the frequency of Foxp3 positive cells in T-cell lymphomas was similar in *HBZ*-Tg mice and in ATL [20]. Second, the suppressive function of Foxp3^+^ T cells was impaired in both ATL and *HBZ*-Tg mice [49]. Third, CD4^+^ T cells in *HBZ*-Tg mice, HTLV-1-infected cells in carriers, and ATL cells possess similar effector/memory and regulatory phenotypes [25], [27]. As shown in this study, transgenic mice expressing Tax under the same promoter as the *HBZ*-Tg mice did not show any changes in the number of Foxp3^+^ T_reg_ cells or effector/memory T cells. These data suggest that HBZ, rather than Tax, is responsible for conferring the specific phenotype of HTLV-1 infected cells and ATL cells.

It has been reported that *tax* transgenic animals develop tumors [50], [51], [52]. In these reports, Tax induced tumors, the type of which depends on the promoter used. However, irrespective of the possible oncogenic activity of Tax, leukemic cells in ATL patients frequently lose Tax expression [7], whereas *HBZ* expression has been detected in all ATL cases studied so far [13]. We reported that the *HBZ* gene transcript itself has growth-promoting activity *in vitro* [13]. Taken together, our results suggest that HBZ is responsible for the specific phenotype, function and proliferation of HTLV-1-infected CD4^+^ T cells and ATL cells, and that HBZ plays important roles for the oncogenic activity of HTLV-1 in addition to Tax. Further, the long latent period before the onset of T-cell lymphomas in *HBZ*-Tg mice suggests that additional genetic and/or epigenetic alterations in CD4^+^ T cells are necessary for the development of T-cell lymphomas in *HBZ*-Tg mice as well as for ATL.

In conclusion, the HBZ-mediated dysregulation of T_reg_ function and proliferation that we report here provides novel insights into the interaction between the host and the virus and may be exploited to treat and prevent HTLV-1-induced diseases.

## Materials and Methods

### Ethics statement

This study was conducted according to the principles expressed in the Declaration of Helsinki. The study was approved by the Institutional Review Board of Kyoto University (E921). All patients provided written informed consent for the collection of samples and subsequent analysis. Animal experimentation was performed in strict accordance with the Japanese animal welfare bodies (Law No. 105 dated 19 October 1973 modified on 2 June 2006), and the Regulation on Animal Experimentation at Kyoto University. The protocol was approved by the Institutional Animal Research Committee of Kyoto University (Permit Number: D09-3). All efforts were made to minimize suffering.

### Mice and cell cultures

C57BL/6J mice were purchased from CLEA Japan. The HBZ cDNA was cloned into the *Sal*I site of the H/M/T-CD4 vector, which was designed for restricted expression of a transgene in CD4^+^ cells. The purified fragment containing the HBZ transgene was microinjected into C57BL/6J F1 fertilized eggs. Transgenic founders were screened for the integration of transgenes in their genomic DNA by PCR and mated with C57BL/6J mice to generate transgenic progeny [13], [15]. All *HBZ*-Tg mice were heterozygotes for the transgene. The phenotype of *HBZ*-Tg mice was stable in the different generations. They express the spliced *HBZ* gene under the control of the *CD4*-specific promoter/enhancer/silencer. All mice were used at 10-16 weeks of age unless specifically described.

The human embryonic kidney cell line, 293FT, was cultured in DMEM containing 10% FCS and G418 (500 µg/ml). The 293FT cell line is derived from the 293F cell line and stably expresses the SV40 large T antigen. 293FT cell line was purchased from Invitrogen. The packaging cell line, Plat-E (kindly provided by T. Kitamura, Tokyo University) was cultured in DMEM supplemented with 10% FCS containing 10 µg/ml blasticidin and 1 µg/ml puromycin. ATL-43T(−) (kindly provided by M. Maeda, Kyoto University) and MT-1 cells were derived from ATL cells, and cultured in RPMI containing 10% FCS and antibiotics (penicillin and streptomycin). A mouse T-cell lymphoma line, EL4 cells, were cultured with RPMI1640 containing 10% FCS, antibiotics, and 50 µM 2-mercaptoethanol (2-ME; Invitrogen).

### Plasmids

In order to construct the vectors expressing tagged spliced HBZ and its mutants, their coding sequences were amplified by PCR, and cloned into the expression vector, pcDNA 3.1(−)/myc-His (Invitrogen). A cDNA clone that contains NFATc2 coding sequence was kindly provided by Kazusa DNA Research Institute. To construct the FLAG-tagged NFATc2 expression vector, its coding region was cloned into pCMV-Tag2 (Stratagene). pCMV-HA (Clontech) was used to generate HA-tagged Foxp3 expression vectors. The vectors expressing Flag-tagged Foxp3 mutants were also used for immunoprecipitation.

### Antibodies and reagents

The following antibodies were used for immunoprecipitation and Western blotting: mouse anti-Flag (clone M2; Sigma, Saint Louis, MO), mouse anti-c-myc (clone 9E10; Sigma), mouse anti-HA (clone HA-7; Sigma), rabbit anti-His polyclonal antibody (MBL), rabbit anti-FOXP3 (polyclonal antibody; Abcam), and rabbit anti-HBZ polyclonal antisera [15].

The following antibodies were purchased from BD PharMingen; purified monoclonal antibody (mAb) for mouse CD4 (RM4-5), CD8α (53-6.7), CD25 (PC61), CD44 (IM7), CD103 (M290), and IL-2 (JES6-5H4). Purified monoclonal antibodies for mouse GITR (DTA-1), CTLA-4 (UC10-4B9), CD62L (MEL-14), TCRβ (H57-597), TCRγδ (eBioGL3) and Foxp3 (FJK-16s) or human FoxP3 (236A/E7) were purchased from eBioscience. Anti-mouse CCR4 antibody (polyclonal antibody; Capralogics) and FITC-labeled anti-goat IgG antibody (Santa Cruz Biotechnology) were used for the detection of mouse CCR4. The following reagents were used for cell culture: anti-CD3ε antibody (145-2C11; R&D systems), Con A (Sigma), PMA (Sigma), and ionomycin (Sigma).

### Synthesis of cDNA and semiquantitative RT-PCR

cDNAs were synthesized from 1 µg total RNA of purified mouse CD4^+^ T cells by a reverse transcriptase SuperScript III and random primers according to the manufacturer's instructions (Invitrogen). Spliced *HBZ* and *GAPDH* transcripts were quantified using RT-PCR. The primers used were as follows: s*HBZ* gene: 5′-TAAACTTACCTAGACGGCGG-3′ (sense), 5′-CTGCCGATCACGATGCGTTT -3′ (antisense); *GAPDH* gene: 5′-GTGGAGA TTGTTGCCATCAACG -3′ (sense) and 5′-AGAGGGGCCATCCACAGTCTT-3′ (antisense). PCR was performed in a PC-808 (Astec) under the following conditions: *HBZ*: 2 minutes at 95°C, followed by 26 cycles of 30 seconds at 95°C, 30 seconds at 59°C and 60 seconds at 72°C; *GAPDH:* 3 minutes at 95°C, followed by 35 cycles of 30 seconds at 95°C, 30 seconds at 61°C and 30 seconds at 72°C.

### Quantitative RT-PCR

To quantify the expression level of *HBZ*, a TaqMan probe and primers for *HBZ* were designed. The sequences of primers and probe for *HBZ* were as follows; *HBZ* primers; 5′-GGACGCAGTTCAGGAGGCAC-3′ (sense) and 5′-CCTCCAAGGATAATAGCCCG-3′ (antisense); *HBZ* probe; 5′-CCTGTGCCATGCCCGGAGGACCTGC-3′. We used the TaqMan Gene expression Assay for *18S rRNA* (Applied Biosystems) as an internal control. Relative expression level of *HBZ* or *IL-2* was calculated with the delta delta Ct method.

### Retroviral constructs and transduction

For retroviral gene transduction experiments, spliced HBZ cDNA was cloned into a retroviral vector, pMXs-Ig (a gift from T. Kitamura), to generate pMXs-Ig-HBZ. pGCSamIN (kindly provided from M. Onodera) and pGCSamIN-Foxp3 were used as previously described. Transfection of the packaging cell line, Plat-E, was performed as described. For retroviral transduction, CD25^−^CD4^+^ cells were enriched by a CD4 enrichment kit (BD Pharmingen) and were activated by 0.5 µg/ml anti-CD3 Ab and 50 U/ml rIL-2 in the presence of T-cell-depleted and x-irradiated (20Gy) C57BL/6J splenocytes as APCs in 12 well plates. After 16 hours, activated T cells were transduced with viral supernatant and 4 µg/ml polybrene, and centrifuged at 3,000 rpm for 60 min. Cells were cultured in medium supplemented with 50 U/ml rIL-2. Activation of naïve T cells by anti-CD3 antibody influenced expression of these molecules. Therefore, we analyzed their expression after influence by activation was lost [35]. Two days later, Foxp3-mediated CTLA-4 expression was detected by a flow cytometry, and five days later, expression of GITR or CD25 was analyzed. After two days, we stimulated the transduced cells with 50 ng/ml PMA and 1 µg/ml ionomycin in the presence of protein transport inhibitor (BD PharMingen) for 6 hours, and then analyzed intracellular IL-2 expression using intracellular cytokine staining kits (BD Pharmingen) according to the manufacturer's instructions.

To elucidate the effect of HBZ on endogenous expression of T_reg_ associated molecules, we transduced HBZ into CD4^+^Foxp3^+^ cells purified from mouse splenocytes. Three days after transduction, the expression levels of T_reg_ associated molecules were evaluated by a flow cytometry.

### Preparation of splenocytes, flow cytometric analyses, cell sorting, and assays of regulatory T cells

Cell suspensions were prepared from murine spleens by forcing the organs through a nylon mesh, and splenic erythrocytes were eliminated with NH_4_Cl. Proliferation of murine cells was measured by ^3^H-thymidine uptake after 3 days of incubation in RPMI1640 medium supplemented with 10% FCS and 50 µM 2-ME. Flow cytometric analyses and cell sorting were carried out using a FACS CantoII or FACS Aria with Diva Software (BD Pharmingen) and the data was analyzed by FlowJo software (Treestar). For cell surface staining, 10^6^ cells were incubated with mAbs for 30 min at 4°C, and then analyzed. For intracellular staining, we used a mouse Foxp3 staining kit according to its protocol (eBioscience). To sort Foxp3^+^ cells, suspended splenocytes were stained with mAb for CD4 and GITR, and the CD4^+^GITR^high^ fraction was sorted by FACS Aria. Purity of the sorted population was always >90% by re-analysis of Foxp3 staining. For the *ex vivo* proliferation assay of Foxp3^+^ cells, carboxy-fluorescein diacetate, succinimidyl ester (CFSE)(Molecular Probe) was used according to the manufacturer's instructions. Foxp3^+^ T cells (2×10^4^/well) were stimulated with anti-CD3 antibody (4 µg/ml) in round-bottomed 96-well plates in the presence of x-irradiated splenocytes as antigen presenting cells (APC; 5×10^4^/well) for 96 hours. Then, cells were permeabilized, and stained with anti-Foxp3. CFSE dilution was analyzed by flow cytometry. To evaluate the suppressive activity of Foxp3^+^ T cells sorted from *HBZ*-Tg or non-Tg mice, Foxp3^+^ T cells (2×10^4^/well) were cultured with CD25^−^CD4^+^ cells (2×10^4^/well) and APCs (5×10^4^/well) from wild-type mice for 72 h in the presence of soluble anti-CD3 (4 µg/ml) or Con A (1 µg/ml), and then [^3^H] thymidine incorporation was measured.

### BrdU staining

*In vivo* proliferation was measured by BrdU incorporation. BrdU (Nacalai Tesque) was dissolved in PBS (3 µg/ml), and then 200 µl was injected intraperitoneally into *HBZ*-Tg and non-transgenic mice twice a day for three days as reported previously [53]. BrdU incorporation in CD4^+^, CD8^+^, or B220^+^ splenocytes was detected using FITC BrdU Flow Kits (BD Pharmingen) according to the manufacturer's instructions. Flow cytometric analyses were performed on a FACS CantoII with Diva Software (BD Pharmingen).

### Foxp3 reporter assay

We constructed Foxp3 promoter and enhancer reporter plasmids as the previous report [34]. A murine T-cell line, EL4 cells (1×10^7^), were transiently cotransfected by electroporation with the following plasmid DNAs: *Foxp3* reporter plasmid, *Renilla* luciferase control vector (pRL-TK), and HBZ expression vector (pME18SneoHBZ). Cells were cultured with or without TGF-β (2 ng/ml). Firefly and *Renilla* luciferase activities were measured using the Dual-Luciferase Reporter Assay System (Promega). Relative luciferase activities were calculated as the ratio of firefly and *Renilla* luciferase activities. The luciferase values are shown as relative values. Values represent means plus standard deviations (error bars) (n = 3).

### Histological analyses

The study of clinical samples was approved by the local research ethics committee of the appropriate hospital. Tissue samples were fixed in 10% formalin in phosphate buffer and then embedded in paraffin. Haematoxylin and eosin (H&E) staining was performed according to standard procedures. Images were captured using a Provis AX80 microscope (Olympus) equipped with OLYMPUS DP70 digital camera, and detected using a DP manager system (Olympus).

For analysis of tumors, mice that became immobilized were sacrificed and subjected to autopsy. Tissue samples were surgically removed and fixed in 10% formalin in phosphate buffer and embedded in paraffin. Sections were stained with H&E for histopathologic examination. After we obtained informed consent, tissue samples from patients who were diagnosed as lymphoma-type ATL were analyzed by immunohistochemical methods to determine FoxP3 expression. Monoclonal antibodies for CD3ε(500A2; BD Pharmingen), B220 (RA3-6B2; BD Pharmingen), and Foxp3 (FJK-16s; eBioscience) were used for immunohistochemistry. We judged CD3^+^B220^+^ cases to be T-cell lymphomas since some activated T cells and T cells of the *lpr/lpr* mutant mouse expressed B220 [54], [55].

### PCR/single stranded conformation polymorphism (SSCP) analysis

To investigate clonality of lymphoma cells observed in *HBZ*-Tg mice, lymphoma tissue samples of *HBZ*-Tg were analyzed for the clonality of T-cell receptor (TCR) γ locus using PCR-SSCP analysis of the TCR γ-gene. Genomic DNA was subjected to PCR amplification using primers for the Vγ2 gene and the Jγ1. The primers used were as follows: Vγ2: 5′-ACCAAGAGATGAGACTGCACAA-3′ (sense), Jγ1: 5′-GCGTCTGATCCTCAAAATAACTTCC-3′ (antisense); PCR was performed in a PC-808 (Astec) under the following conditions: 3 minutes at 95°C, followed by 35 cycles of 30 seconds at 95°C, 30 seconds at 55°C and 30 seconds at 72°C. We used EL-4 as a positive control and splenic DNA from young non-Tg or *HBZ*-Tg mice as negative control. PCR products were run on a 6% polyacrylamide gel and visualized by staining with DNA Silver Staining Kit (GE Healthcare).

### Coimmunoprecipitation assay and immunoblotting

Expression vectors for the relevant genes were transiently cotransfected into 293FT cells using the TransIT-LT1 reagent (Mirus Bio). 24 hours later, transfected cells were stimulated with 50 ng/ml PMA and 1 µg/ml ionomycin for another 6 hours. Coimmunoprecipitation assays were performed using the Nuclear Complex Co-IP Kit (Active motif). Briefly, the nuclear extracts of transfected cells were prepared in the presence or absence of ethidium bromide (10 µg/ml). They were precleared with Protein G Sepharose 4 Fast Flow (GE Healthcare), and their supernatants were incubated with anti-myc tag (clone 9E10, Sigma) or anti-Flag tag (M2, Sigma) antibody overnight at 4°C. The immunocomplexes were precipitated with Protein G Sepharose 4 Fast Flow, fractionated in SDS-PAGE, and transferred to PVDF membranes. HBZ-myc-His was detected with horseradish peroxidase (HRP)-conjugated anti-His tag (MBL) antibody. HRP-conjugated anti-Flag tag (Sigma) and anti-HA tag (Sigma) antibodies were used to detect Flag-tagged and HA-tagged proteins, respectively. To detect endogenous interaction between HBZ and FoxP3, immunoprecipitation was performed using an ATL cell line, ATL-43T(-), as described above with anti-HBZ antisera and anti-FOXP3 antibody (Abcam). To examine the expression of HBZ in transgenic mice, CD4^+^ splenocytes from wild type or *HBZ*-Tg mice were enriched by a mouse CD4 T lymphocyte enrichment set (Pharmingen). Whole cell extracts were prepared with the lysis buffer (50 mM Tris-HCL, PH 7.5, 150 mM NaCl, 1% NP-40), and analyzed by western blotting with anti-HBZ antisera.

### Flow cytometric analysis for HTLV-1 carrier cells

A previous report demonstrated that Tax expression could not be detected in freshly isolated PBMC from HTLV-1 infected carriers but could be detected when they were cultivated *ex vivo* for 12 hours [56]. We cultured PBMCs from asymptomatic HTLV-1 carriers for 12 hours and stained with monoclonal antibodies against FoxP3 or Tax (MI-73), and then analyzed by flow cytometry.

### Statistical analysis

For *in vitro* experiments, multiple data comparisons were performed using Student's unpaired *t*-test. Statistical differences in the incidence of T-cell lymphoma were analyzed using a logrank test.

## Supporting Information

Figure S1 Characterization of the transgene. (A) Schematic structure of the transgene. (B) Copy numbers of the transgene in each line were determined by Southern blot analysis. Serially diluted plasmids, used to calculate the copy number, are shown on the left side.
(0.35 MB TIF)

Figure S2 Histological analysis of the skin and lung of *HBZ*-Tg mice. HE staining showed massive infiltration of lymphocytes in *HBZ*-Tg line 9 and 12, but not in line 2. Immunohistochemical staining revealed that only some of infiltrating lymphocytes were FoxP3 positive. Arrows indicate FoxP3 positive cells.
(4.46 MB TIF)

Figure S3 Flow cytometric analysis of TCRβ and TCR γδ expression in the spleen with lymphoma observed in *HBZ*-Tg mice. Numbers are identical to those of Table 1.
(0.32 MB TIF)

Figure S4 PCR/single stranded conformation polymorphism (SSCP) analysis. *HBZ*-Tg lymphoma tissue samples were analyzed for TCR clonality using PCR-SSCP analysis of the TCR γ-gene. EL-4 are shown as a positive control and splenic DNA from young (less than 6 weeks old) non-Tg or *HBZ*-Tg mice as a negative control. Lanes 1- 5 (#2-3, #9-1, #12-6, #9-3, #12-7) show lymphoma from *HBZ*-Tg mice respectively (Table 1).
(1.09 MB TIF)

Figure S5 Analysis of FoxP3 expression in fresh ATL cells. Immunohistochemical staining for FoxP3 in the lymph nodes of human ATL patients. We used a monoclonal antibody for human FoxP3 (236A/E7; eBioscience).
(1.89 MB TIF)

Figure S6 Flow cytometric analysis of thymocyte subsets. Non-Tg or *HBZ*-Tg thymocytes were stained with anti-CD4 and anti-CD8 antibody, and then analyzed by flow cytometry.
(0.48 MB TIF)

Figure S7 Foxp3 expression in spleen, cervical lymph node, or peripheral blood mononuclear cells was determined by flow cyotmetry. Representative histograms gated on the CD4^+^ population are shown.
(0.36 MB TIF)

Figure S8 *HBZ*-Tg line 2 also showed an increase in effector/memory and regulatory CD4 T cells. Mouse splenocytes were stained with antibodies for CD4 and CD8 plus CD44 and CD62L (A) or CD25 and Foxp3 (B), and then analyzed by flow cytometry. Representative dot plots gated on the CD4^+^ population are shown.
(0.57 MB TIF)

Figure S9 IL-2 production of CD4^+^ T cells in *HBZ*-Tg mice. (A) Mouse splenocytes were stimulated with Leukocyte Activation Cocktail, which contains PMA/Ionomycin and protein transport inhibitor (BD Pharmingen), for 4 hours and then analyzed for intracellular IL-2 gated on the CD4^+^ cells by flow cytometry. Representative results of more than three independent experiments are shown. (B) The percentage of IL-2^+^ cells among Foxp3^+^ cells is shown. The results shown are the mean ± SD of triplicate experiments.
(0.26 MB TIF)

Figure S10 Flow cytometric analyses of *tax*-Tg mice. Non-Tg or *tax*-Tg splenocytes were stained with the indicated antibodies, and analyzed by flow cytometry. Representative dot plots gated on the CD4^+^ population are shown.
(0.54 MB TIF)

Figure S11 The effect of HBZ on Foxp3/AML-1 complex. (A) 293FT-cells were co-transfected with vectors expressing the indicated proteins, lysed, and subjected to immunoprecipitation. (B) Jurkat cells were co-transfected with expression vectors for the indicated proteins and IL-2 promoter-luc constructs. The results shown are relative values of firely luciferase normalized to Renilla luciferase and expressed as means ± SD. The experiments were repeated three times with similar results.
(0.37 MB TIF)

Figure S12 Characterization of the interaction between HBZ and NFAT. To investigate the region responsible for each interaction, we performed immunoprecipitation experiments with NFATC2 and deletion mutants of HBZ. Asterisk shows the region responsible for the molecular interaction.
(0.25 MB TIF)

Figure S13 The percentages of HTLV-1^+^ T cells in CD4^+^FoxP3^−^ and CD4^+^FoxP3^+^ subpopulations of asymptomatic HTLV-1 carriers. It has been reported that ex vivo culture induces the reactivation of viral antigen in HTLV-1 infected cells. We cultured freshly isolated PBMC from two asymptomatic HTLV-1 carriers for 18 hours, and then stained intracellular Tax as a viral antigen to detect the presence of HTLV-1 by using a monoclonal antibody of Tax (MI-73).
(0.31 MB TIF)

Table S1 (A) Summary of BrdU incorporation *in vivo*. Data shown are percentage of BrdU positive cells of three different non-Tg or *HBZ*-Tg mice. (B) MFI of Treg associated molecules (CTLA-4, GITR, CD103, or CCR4) in non-Tg or *HBZ*-Tg (line 12) mice are shown as mean ± SD (n = 3). of three mice. *, P<0.05; **, P<0.01 by two-tailed Student t-test.
(0.25 MB TIF)
