# Supplementary figures and images for: HTLV-1 bZIP Factor Induces T-Cell Lymphoma and Systemic Inflammation In Vivo
Source: PLoS Pathog. 2011 Feb 10;7(2):e1001274. doi: 10.1371/journal.ppat.1001274 (PMC3037353; doi:10.1371/journal.ppat.1001274)

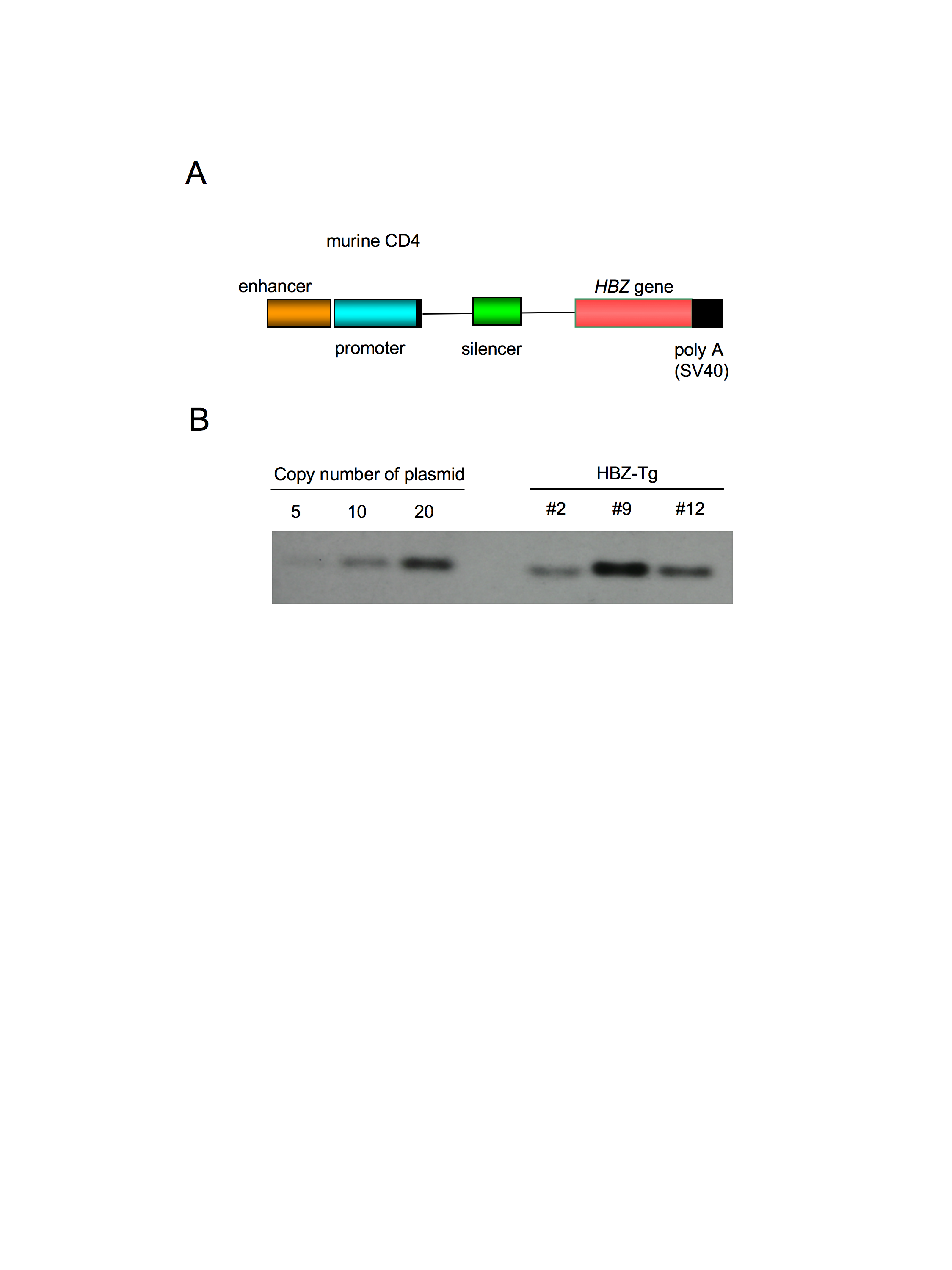

Supplement: Figure S1 — Characterization of the transgene. (A) Schematic structure of the transgene. (B) Copy numbers of the transgene in each line were determined by Southern blot analysis. Serially diluted plasmids, used to calculate the copy number, are shown on the left side. (0.35 MB TIF) [file ppat.1001274.s001.tif]

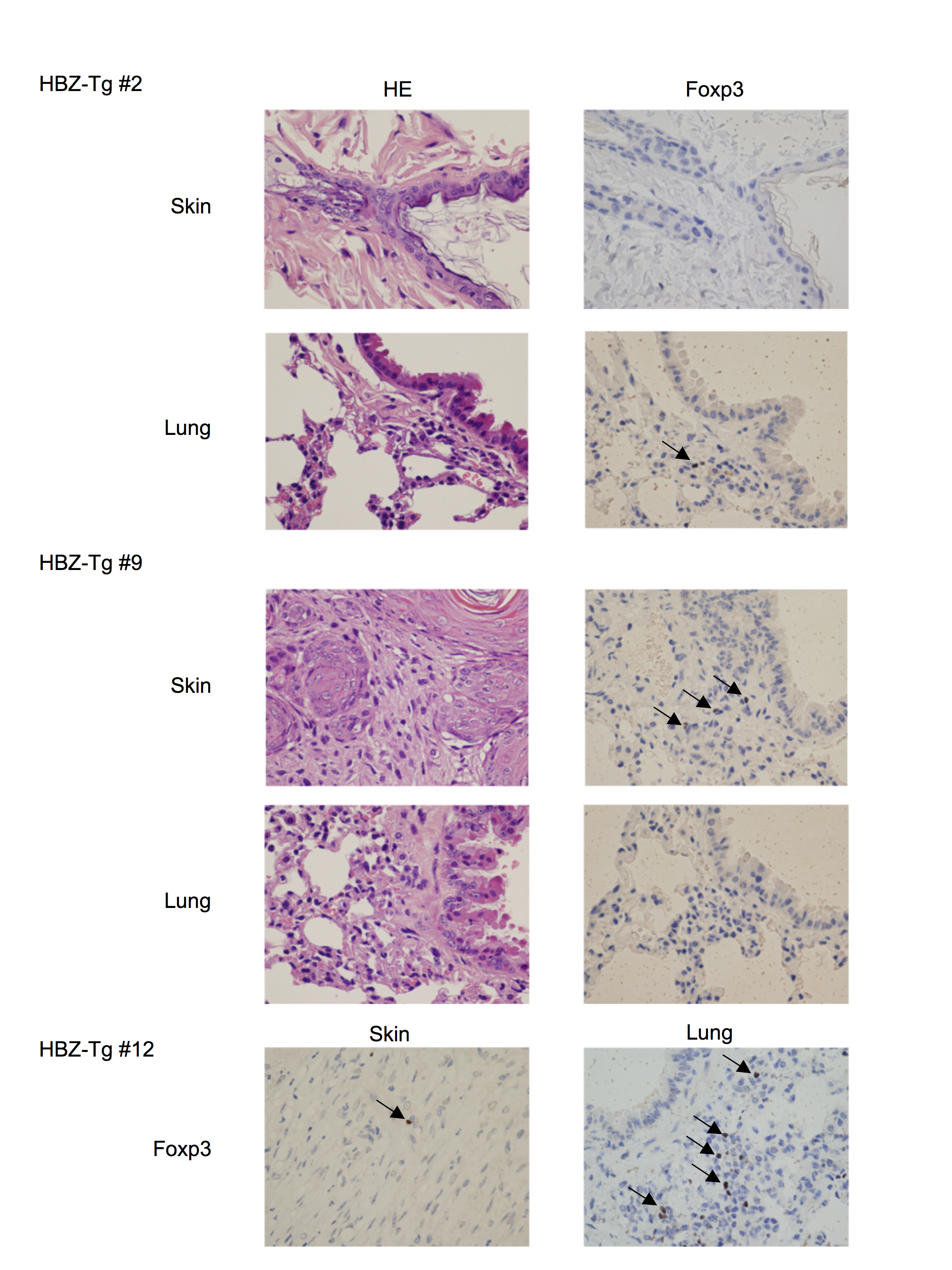

Supplement: Figure S2 — Histological analysis of the skin and lung of HBZ-Tg mice. HE staining showed massive infiltration of lymphocytes in HBZ-Tg line 9 and 12, but not in line 2. Immunohistochemical staining revealed that only some of infiltrating lymphocytes were FoxP3 positive. Arrows indicate FoxP3 positive cells. (4.46 MB TIF) [file ppat.1001274.s002.tif]

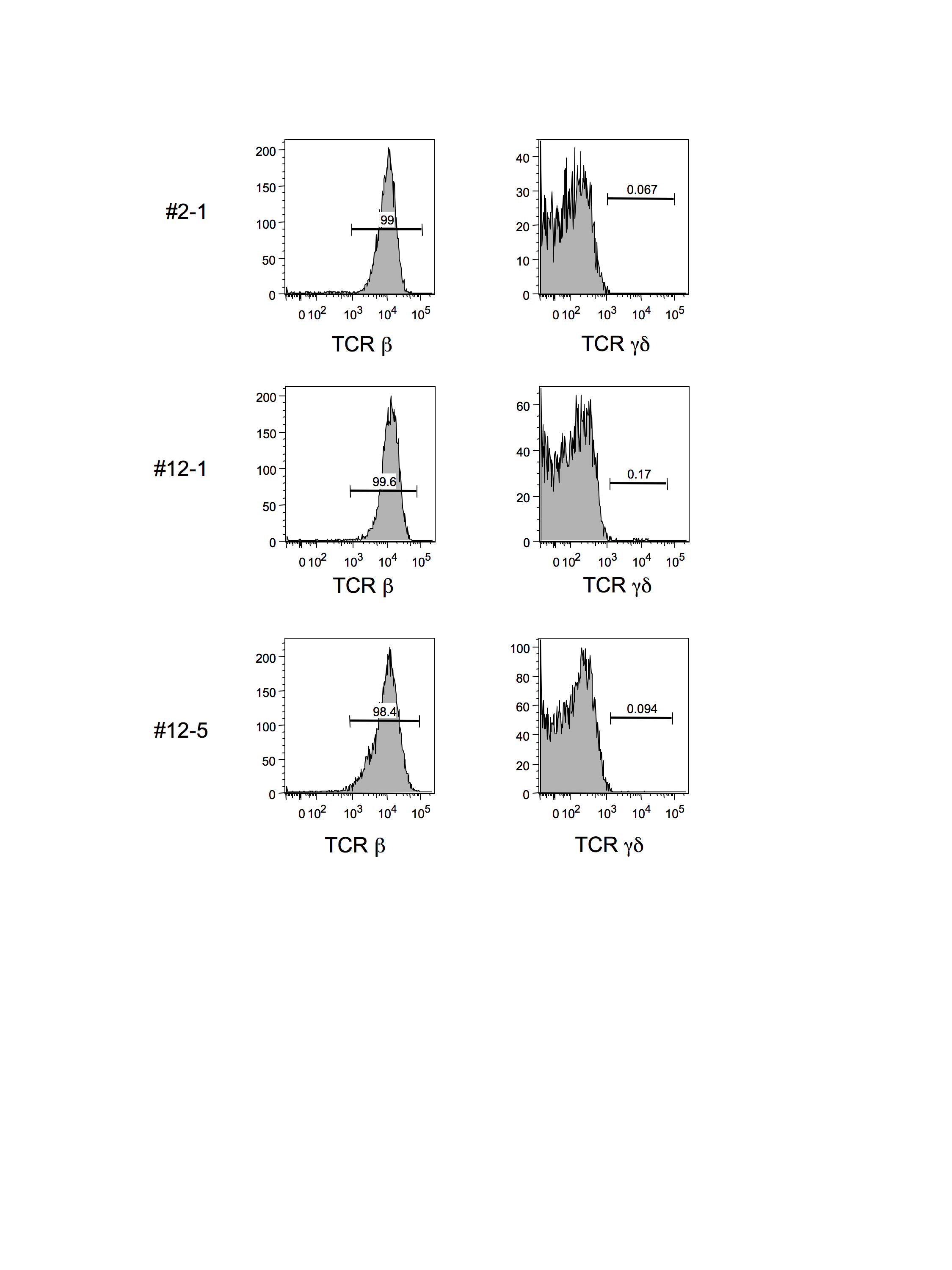

Supplement: Figure S3 — Flow cytometric analysis of TCRβ and TCR γδ expression in the spleen with lymphoma observed in HBZ-Tg mice. Numbers are identical to those of Table 1. (0.32 MB TIF) [file ppat.1001274.s003.tif]

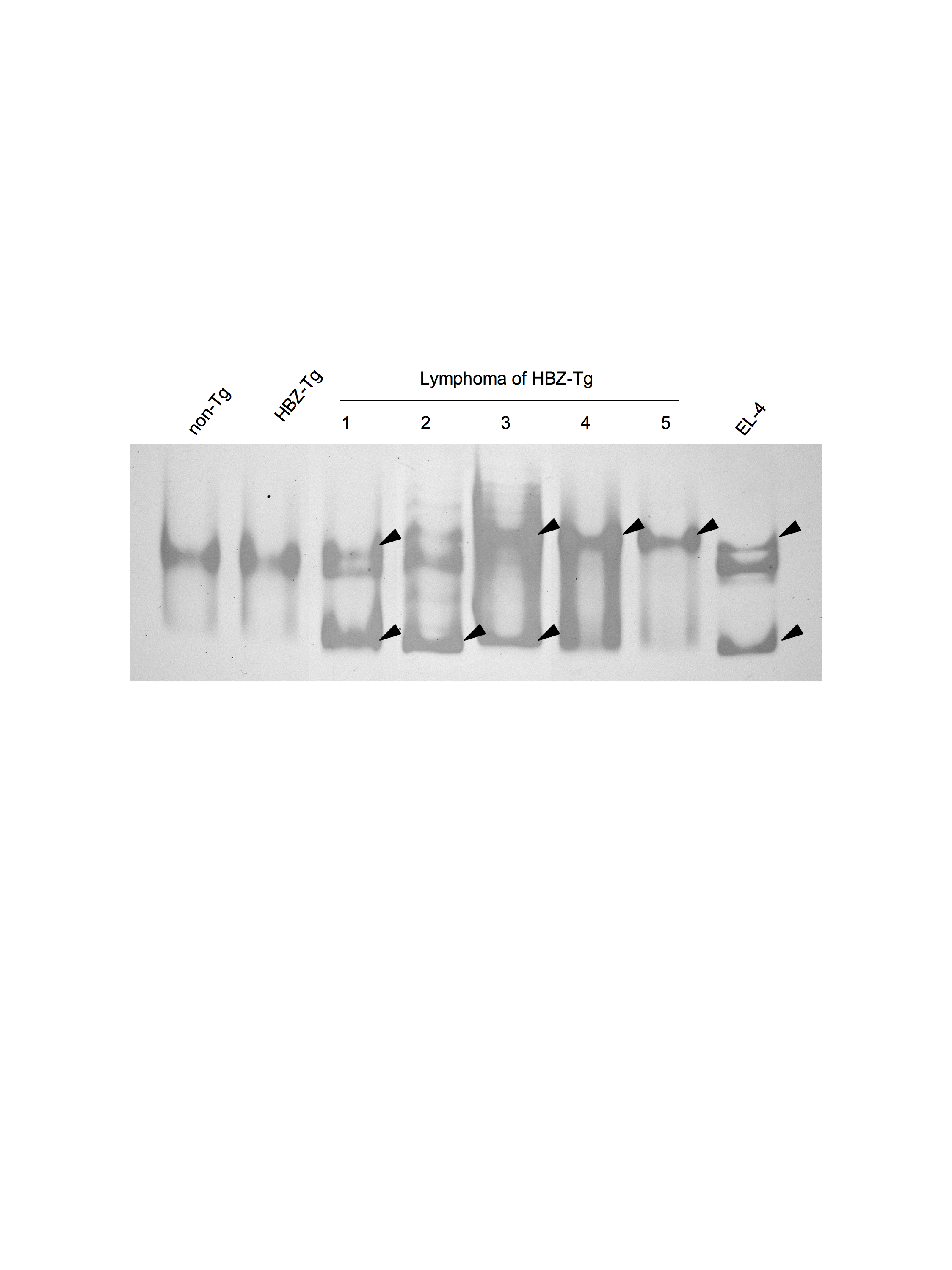

Supplement: Figure S4 — PCR/single stranded conformation polymorphism (SSCP) analysis. HBZ-Tg lymphoma tissue samples were analyzed for TCR clonality using PCR-SSCP analysis of the TCR γ-gene. EL-4 are shown as a positive control and splenic DNA from young (less than 6 weeks old) non-Tg or HBZ-Tg mice as a negative control. Lanes 1- 5 (#2-3, #9-1, #12-6, #9-3, #12-7) show lymphoma from HBZ-Tg mice respectively (Table 1). (1.09 MB TIF) [file ppat.1001274.s004.tif]

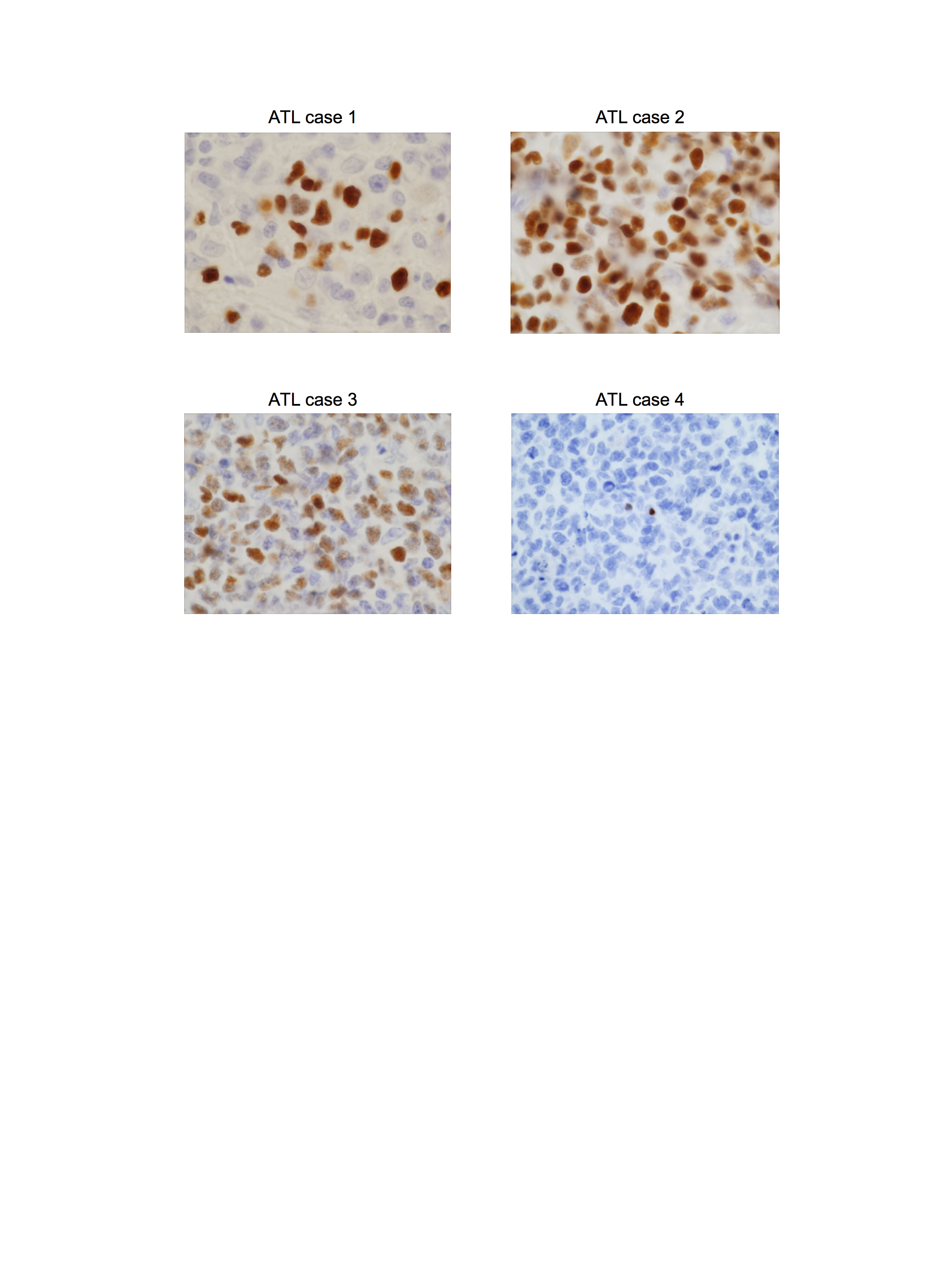

Supplement: Figure S5 — Analysis of FoxP3 expression in fresh ATL cells. Immunohistochemical staining for FoxP3 in the lymph nodes of human ATL patients. We used a monoclonal antibody for human FoxP3 (236A/E7; eBioscience). (1.89 MB TIF) [file ppat.1001274.s005.tif]

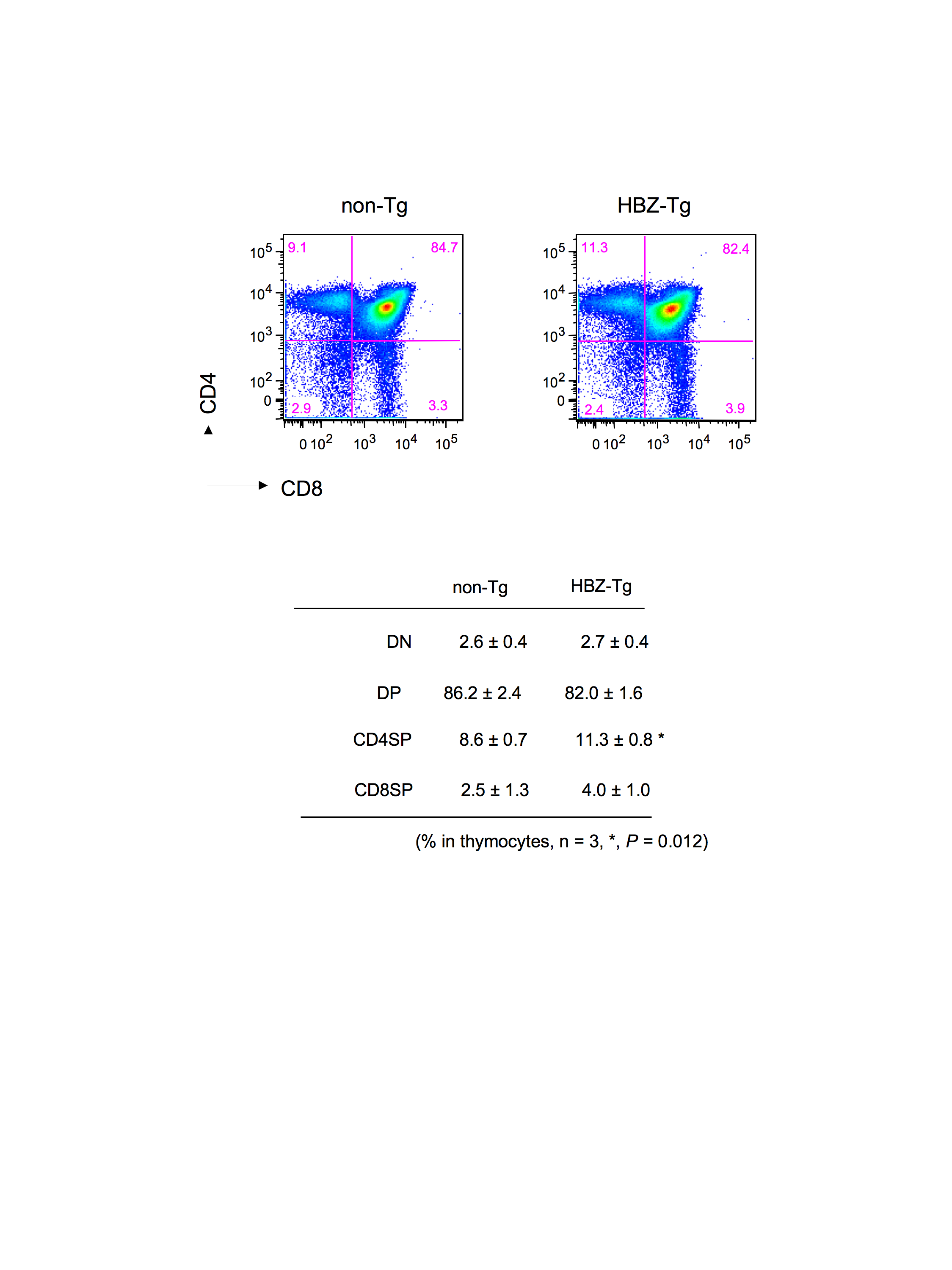

Supplement: Figure S6 — Flow cytometric analysis of thymocyte subsets. Non-Tg or HBZ-Tg thymocytes were stained with anti-CD4 and anti-CD8 antibody, and then analyzed by flow cytometry. (0.48 MB TIF) [file ppat.1001274.s006.tif]

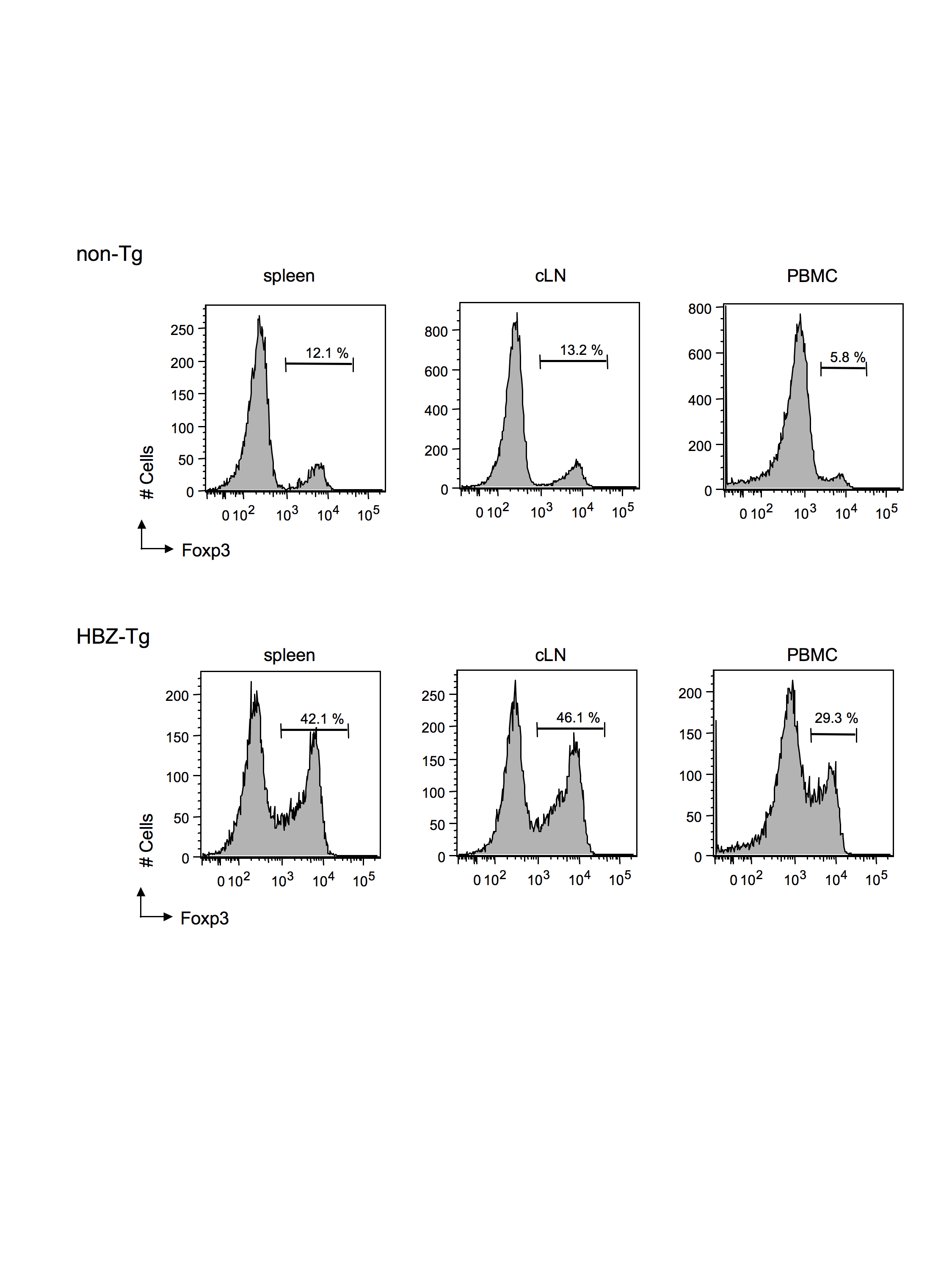

Supplement: Figure S7 — Foxp3 expression in spleen, cervical lymph node, or peripheral blood mononuclear cells was determined by flow cyotmetry. Representative histograms gated on the CD4+ population are shown. (0.36 MB TIF) [file ppat.1001274.s007.tif]

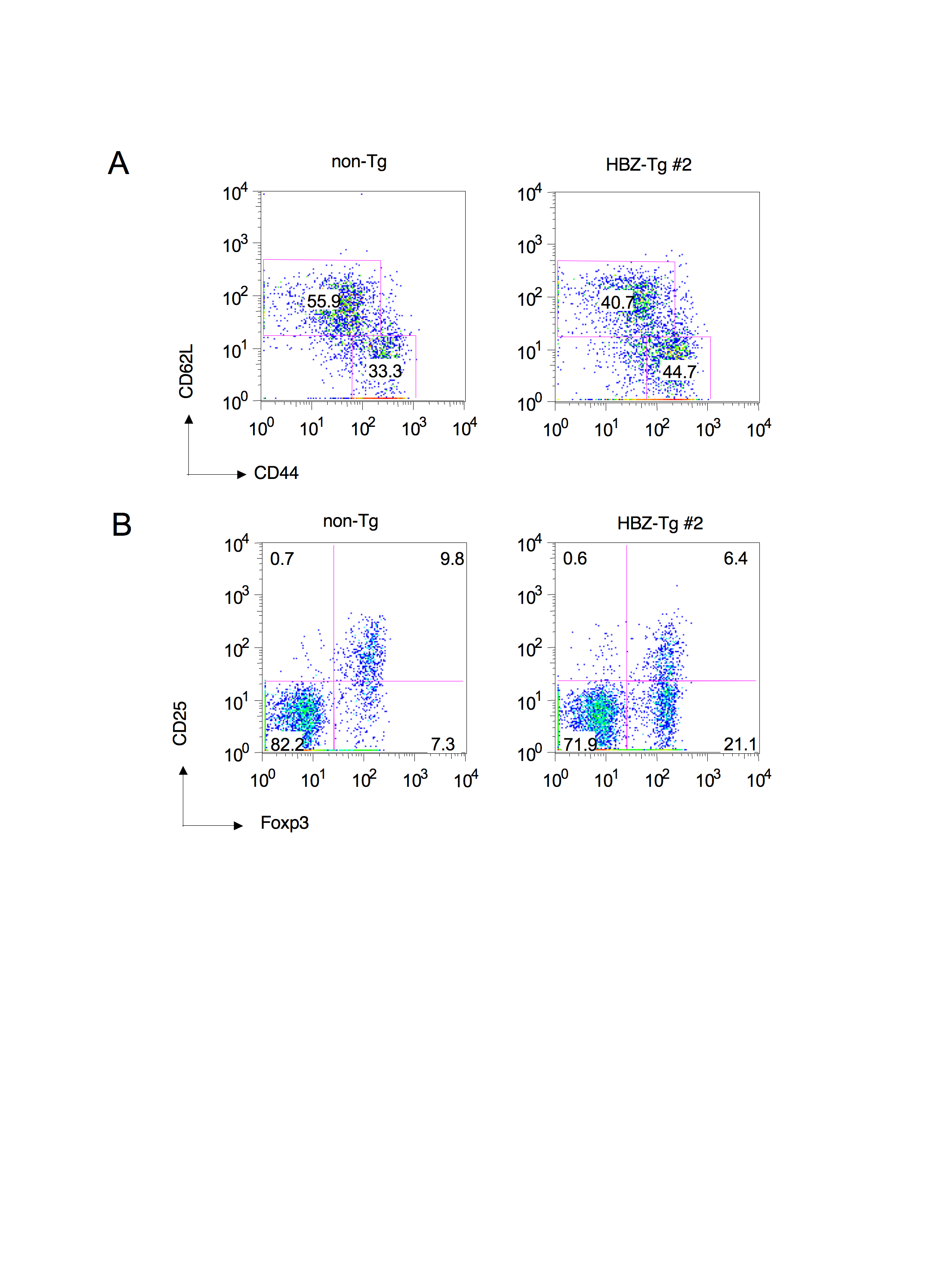

Supplement: Figure S8 — HBZ-Tg line 2 also showed an increase in effector/memory and regulatory CD4 T cells. Mouse splenocytes were stained with antibodies for CD4 and CD8 plus CD44 and CD62L (A) or CD25 and Foxp3 (B), and then analyzed by flow cytometry. Representative dot plots gated on the CD4+ population are shown. (0.57 MB TIF) [file ppat.1001274.s008.tif]

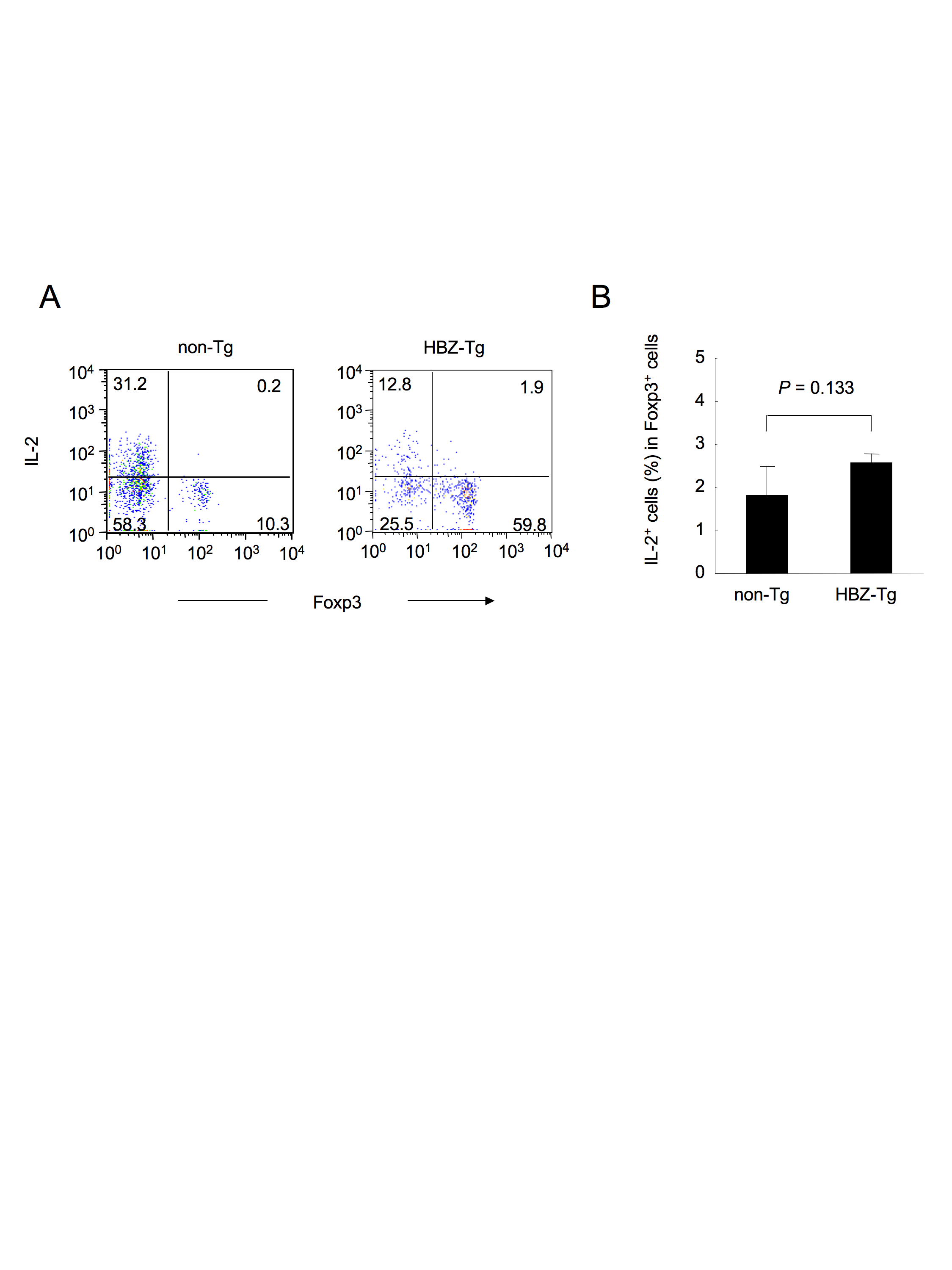

Supplement: Figure S9 — IL-2 production of CD4+ T cells in HBZ-Tg mice. (A) Mouse splenocytes were stimulated with Leukocyte Activation Cocktail, which contains PMA/Ionomycin and protein transport inhibitor (BD Pharmingen), for 4 hours and then analyzed for intracellular IL-2 gated on the CD4+ cells by flow cytometry. Representative results of more than three independent experiments are shown. (B) The percentage of IL-2+ cells among Foxp3+ cells is shown. The results shown are the mean ± SD of triplicate experiments. (0.26 MB TIF) [file ppat.1001274.s009.tif]

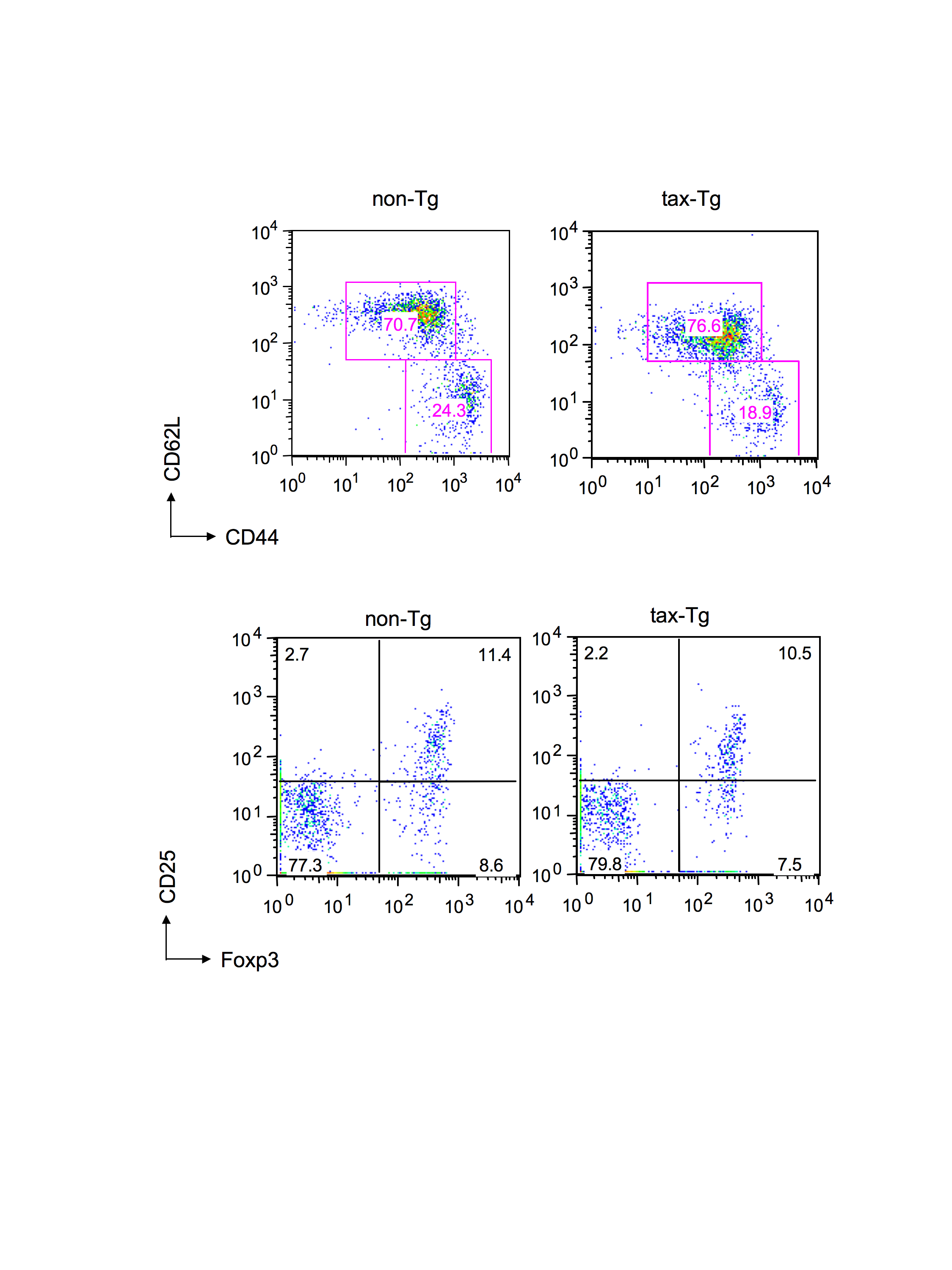

Supplement: Figure S10 — Flow cytometric analyses of tax-Tg mice. Non-Tg or tax-Tg splenocytes were stained with the indicated antibodies, and analyzed by flow cytometry. Representative dot plots gated on the CD4+ population are shown. (0.54 MB TIF) [file ppat.1001274.s010.tif]

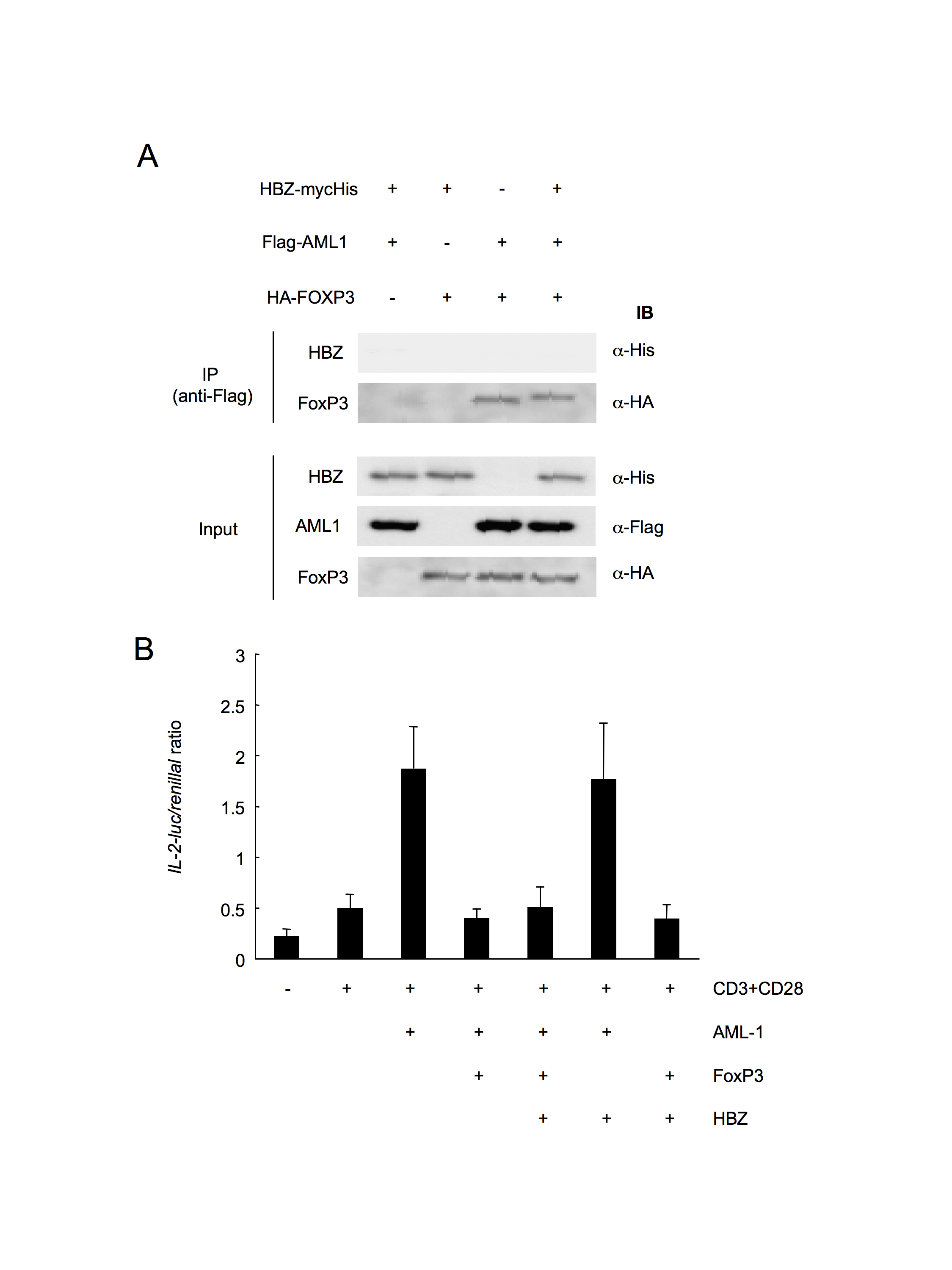

Supplement: Figure S11 — The effect of HBZ on Foxp3/AML-1 complex. (A) 293FT-cells were co-transfected with vectors expressing the indicated proteins, lysed, and subjected to immunoprecipitation. (B) Jurkat cells were co-transfected with expression vectors for the indicated proteins and IL-2 promoter-luc constructs. The results shown are relative values of firely luciferase normalized to Renilla luciferase and expressed as means ± SD. The experiments were repeated three times with similar results. (0.37 MB TIF) [file ppat.1001274.s011.tif]

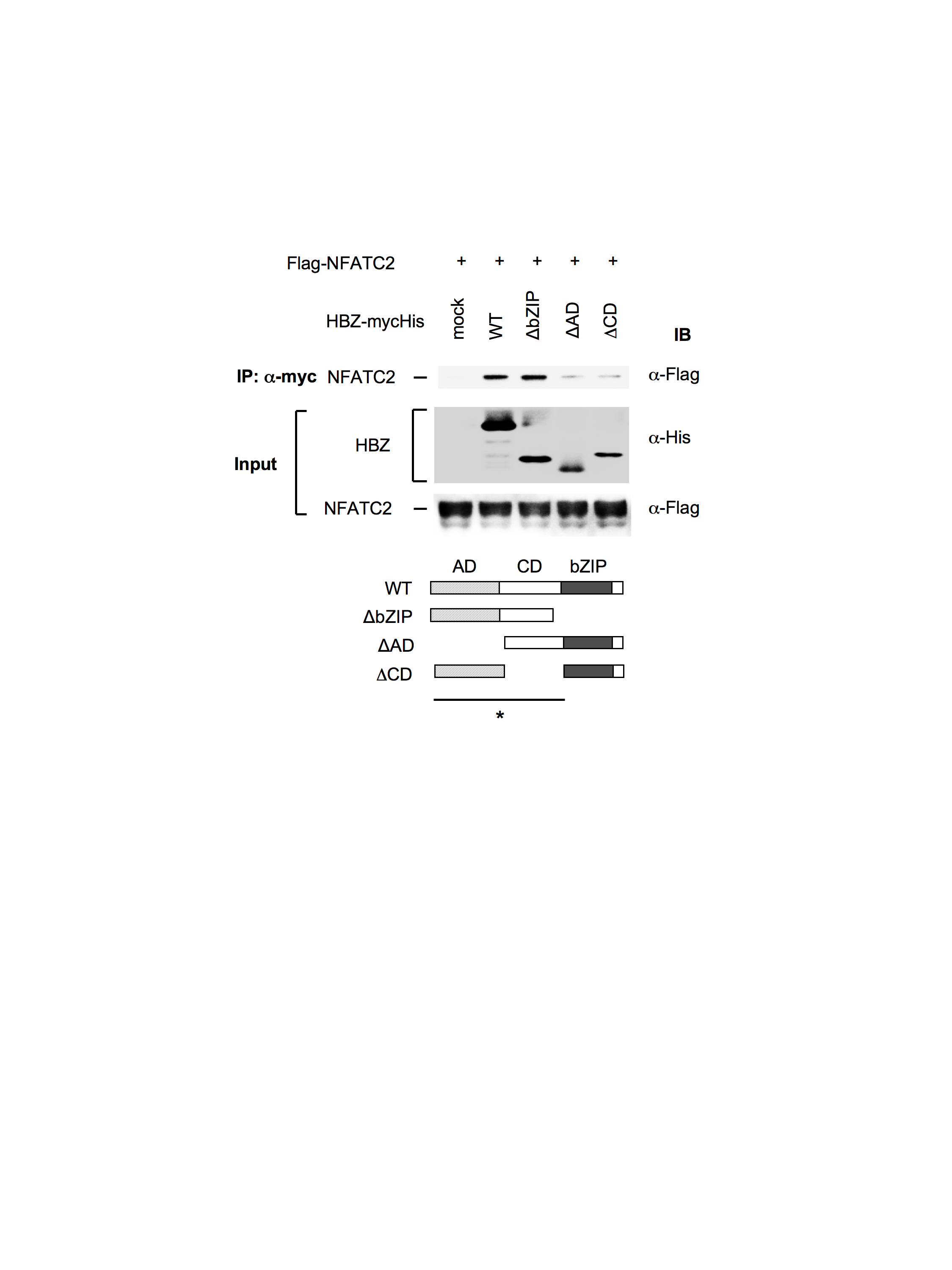

Supplement: Figure S12 — Characterization of the interaction between HBZ and NFAT. To investigate the region responsible for each interaction, we performed immunoprecipitation experiments with NFATC2 and deletion mutants of HBZ. Asterisk shows the region responsible for the molecular interaction. (0.25 MB TIF) [file ppat.1001274.s012.tif]

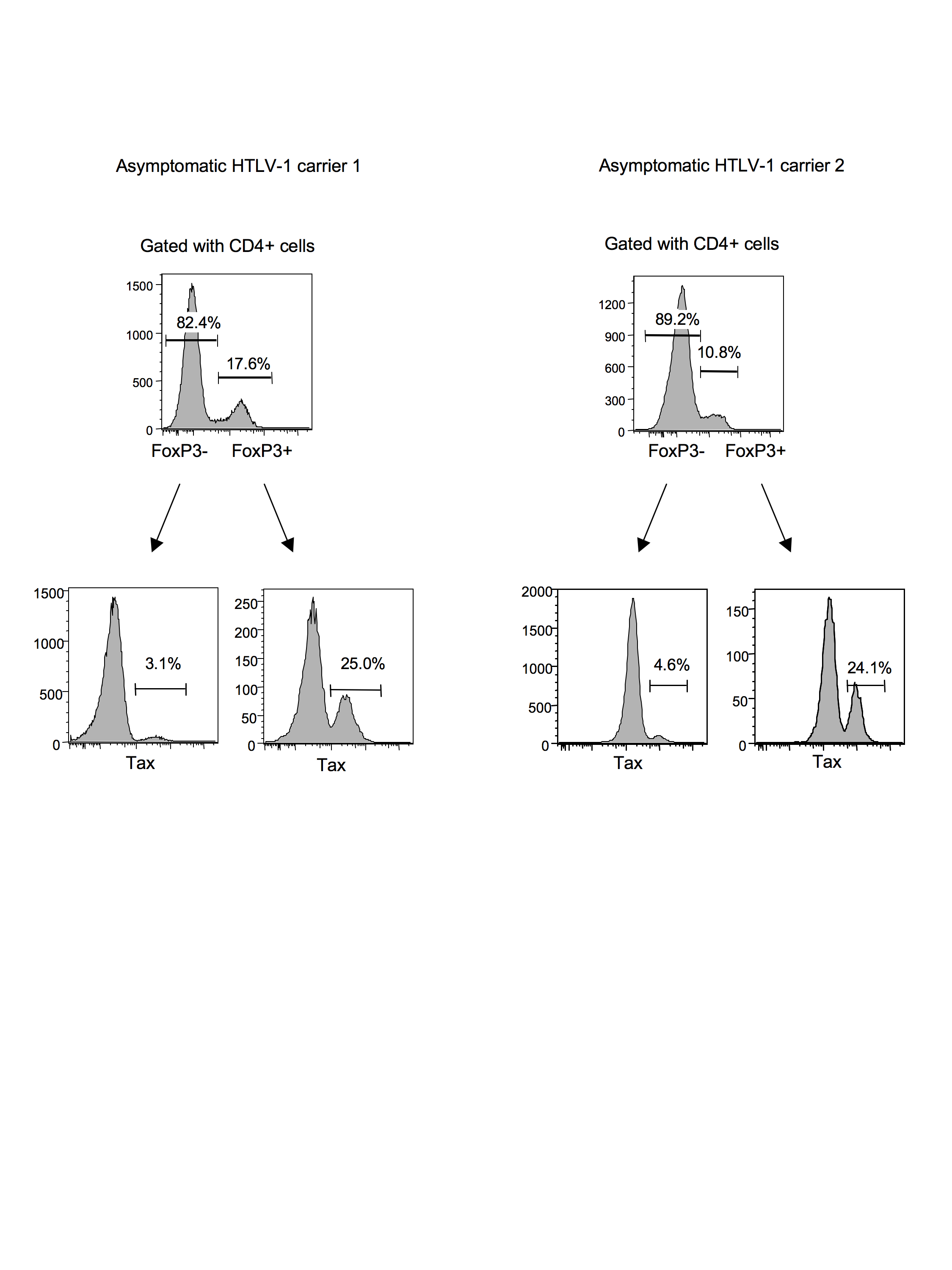

Supplement: Figure S13 — The percentages of HTLV-1+ T cells in CD4+FoxP3− and CD4+FoxP3+ subpopulations of asymptomatic HTLV-1 carriers. It has been reported that ex vivo culture induces the reactivation of viral antigen in HTLV-1 infected cells. We cultured freshly isolated PBMC from two asymptomatic HTLV-1 carriers for 18 hours, and then stained intracellular Tax as a viral antigen to detect the presence of HTLV-1 by using a monoclonal antibody of Tax (MI-73). (0.31 MB TIF) [file ppat.1001274.s013.tif]

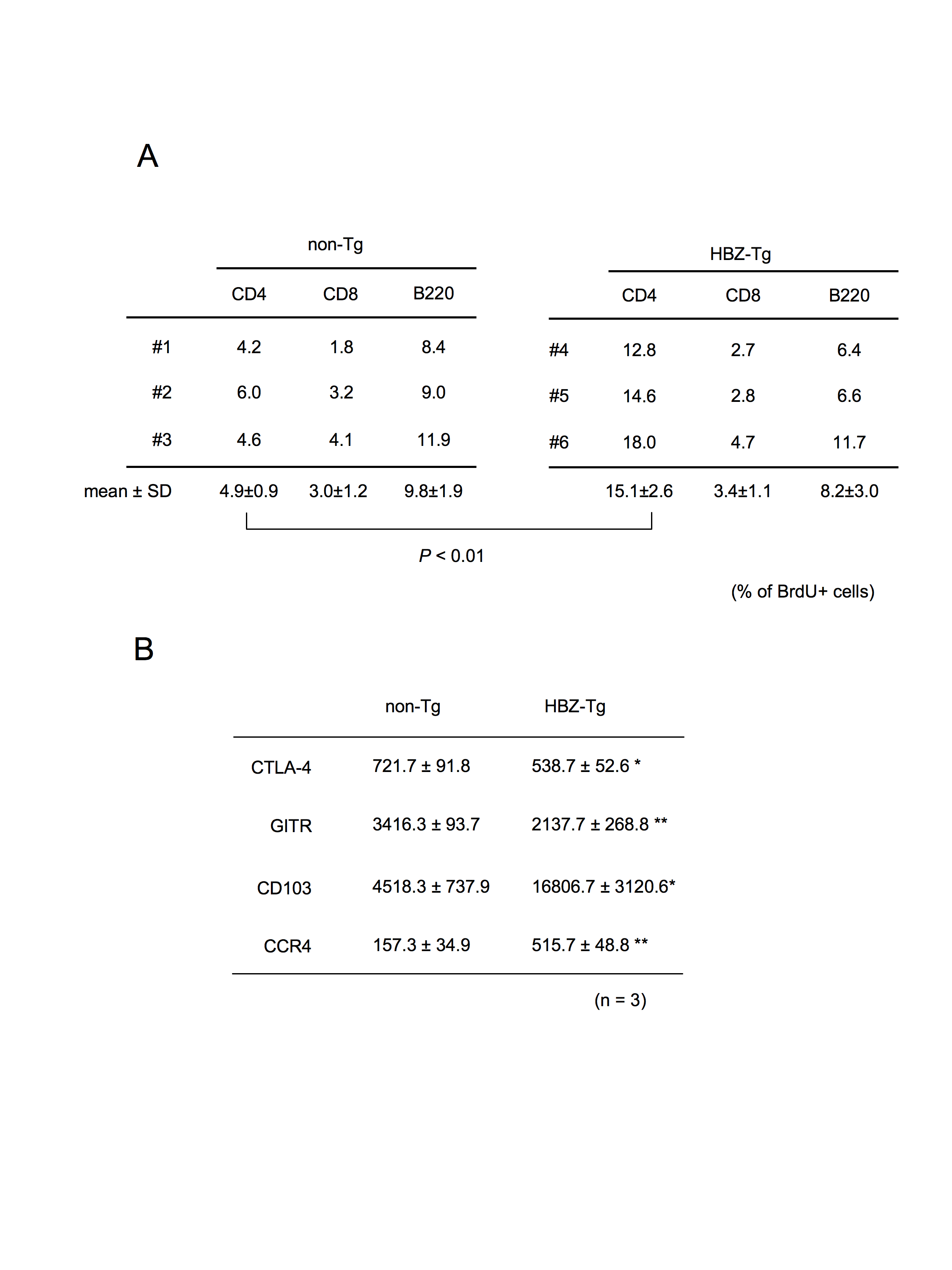

Supplement: Table S1 — (A) Summary of BrdU incorporation in vivo. Data shown are percentage of BrdU positive cells of three different non-Tg or HBZ-Tg mice. (B) MFI of Treg associated molecules (CTLA-4, GITR, CD103, or CCR4) in non-Tg or HBZ-Tg (line 12) mice are shown as mean ± SD (n = 3). of three mice. *, P<0.05; **, P<0.01 by two-tailed Student t-test. (0.25 MB TIF) [file ppat.1001274.s014.tif]
